# Long-term consistency of aperiodic and periodic physiomarkers in subthalamic local field potentials in Parkinson’s disease

**DOI:** 10.1038/s41531-025-01053-5

**Published:** 2025-07-10

**Authors:** Mariëlle J. Stam, Bernadette C. M. van Wijk, Arthur W. G. Buijink, Dan Piña-Fuentes, Rob M. A. de Bie, Rick Schuurman, Martijn Beudel

**Affiliations:** 1 Department of Neurology, Amsterdam UMC, University of Amsterdam, Amsterdam Neuroscience, Amsterdam, the Netherlands; 2 Department of Human Movement Sciences, Faculty of Behavioural and Movement Sciences, Vrije Universiteit Amsterdam, Amsterdam, the Netherlands; 3 Department of Neurosurgery, Amsterdam UMC, University of Amsterdam, Amsterdam Neuroscience, Amsterdam, the Netherlands

**Keywords:** Parkinson's disease, Neurophysiology

## Abstract

Beta oscillations (±13–35 Hz) and aperiodic spectral features extracted from local field potential (LFP) recordings have been identified as promising physiomarkers for adaptive deep brain stimulation (aDBS) in Parkinson’s disease. However, the long-term consistency of these signal features across behavioural and clinical conditions remains unclear. Bilateral subthalamic nucleus LFPs were recorded from twelve patients with an average inter-recording interval of 137 days, during rest, a finger-to-nose task and speech, with stimulation switched off and on. Intra-class correlation coefficients indicated moderate between-visit consistency for aperiodic offset and exponent but good to excellent consistency of beta peak power. Most aperiodic and power changes induced by task execution and stimulation were statistically comparable across visits. Results remained inconclusive regarding the properties of beta peaks exhibiting the strongest power suppression post-stimulation. Our findings support the potential of beta peak power as primary physiomarker for aDBS, with aperiodic components as possibly suitable supplementary markers.

## Introduction

Deep brain stimulation (DBS) has become a standard treatment for selected patients with Parkinson’s disease (PD) who experience motor complications^1,2^. However, current DBS technology does not adjust the stimulation to daily fluctuations in the clinical state of PD, which may result in suboptimal clinical outcomes and side effects^3^. Adaptive DBS (aDBS) technology has emerged as a promising approach to overcome these limitations by using input from neural recordings or other sources to adjust stimulation parameters in real-time^3–5^. A crucial aspect of aDBS is finding an input signal that best represents the clinical state of a patient, a so-called *physiomarker*. Local field potential (LFP) signals recorded with the implanted DBS electrodes can be used for obtaining such physiomarkers.

LFP signals are typically characterized by periodic and aperiodic components, which can be separated in the frequency domain. The periodic component consists of rhythmic neural activity, of which the spectral power of beta band oscillations (±13–35 Hz) in the subthalamic nucleus (STN) and internal pallidum (GPi) has been consistently linked to motor symptoms and levodopa responsiveness^6–10^. Both upper^11–13^ and lower^14^ limb movement as well as the administration of high frequency (~130 Hz) DBS^15–17^ have been shown to attenuate LFP beta band power, whereas for speech both a decrease^18–20^ and increase^21^ of beta power has been reported. Other periodic components that may serve as physiomarkers include the presence of finely-tuned gamma oscillations, associated with the medicated ON state^11^ and treatment-induced dyskinesias^22^, and low frequency oscillations measured in the alpha or theta band that have been related to mood and cognitive states^23^.

The aperiodic component, also referred to as the 1/f-component, consists of the exponential decrease of power with increasing frequency^24^. It can be modeled with an “offset” parameter, reflecting the broadband shift in power across frequencies, as well as an “exponent” parameter, reflecting the negative slope of the power spectrum when measured in log-log space^25,26^. The aperiodic exponent is associated with the balance between excitatory and inhibitory synaptic inputs (E/I) to the site of recording^27^. The loss of dopaminergic neurons in PD leads to a reduced inhibition of the STN, which is alleviated with dopaminergic medication and stimulation^28,29^. Accordingly, the aperiodic exponent of electrophysiological spectra from the STN has been found to increase with dopaminergic medication and DBS, and has therefore also been suggested as potential physiomarker^30^. Indeed, the aperiodic exponent was shown to be both informative of motor symptom severity as well as predictive of the response to DBS^31^.

For an effective aDBS system that automatically adjusts stimulation parameters according to the recorded signals, changes in physiomarkers should reflect variations in the patient’s clinical state, rather than being purely triggered by movement- or stimulation-induced effects on the LFP features. For example, movement-induced beta power suppression has been shown to reduce responsivity of beta-based aDBS algorithms^32–34^, however stimulation should ideally not be interrupted when a patient starts moving. Similarly, the aDBS algorithm should be responsive to a physiomarker that consistently reflects the variations in a patient’s clinical state as a result of stimulation, rather than respond to the changes in LFP features caused by the stimulation itself. As yet, it is unknown whether distinguishing between patient’ states for aDBS is best performed according to absolute values of LFP features or by taking a baseline condition as a reference (e.g., values relative to rest with stimulation OFF). It is not only important that physiomarkers are indicative of the clinical state of the patient, but also that the association is stable across a time period of several weeks or months until at least the next routine hospital visit when stimulation parameters may be further refined depending on the experienced effectiveness of the aDBS algorithm. Assessing the consistency of multiple LFP features under different behavioral and clinical conditions allows for comparing which physiomarkers are resilient to natural fluctuations over time and hence might remain effective over long periods. Furthermore, quantifying the natural variability helps to determine whether fixed thresholds for stimulation can be used reliably or if dynamic adjustments are required to maintain effectiveness.

So far, scientific understanding of potential physiomarkers for PD has been primarily gained from intra-operative or immediate postoperative neural recordings using externalized leads. Initial studies particularly analyzed the peak frequency and (change of) power of beta oscillations after initial implantation^17,35^. Later studies also recorded beta oscillations during battery replacement surgery, and showed that beta band power stayed almost unchanged for years after DBS electrode implantation^36^. First-generation fully implanted bidirectional neurostimulators allowed for investigating physiomarkers at any given time after DBS implantation^37,38^, but were only available for investigational purposes and recordings contained strong interference from stimulation artifacts^39^. In 2020, a second-generation fully implanted neurostimulator with sensing capabilities, the Medtronic Percept^TM^, became commercially available thereby aiding the development of aDBS. Apart from a recent study demonstrating beta peak power consistency in the first months after initial DBS implantation^40^, it is still unclear how other physiomarkers derived from the periodic and aperiodic components and their responsiveness to movement and stimulation behave over the course of several months or years after the implantation.

In this study we aimed to establish the consistency of beta band features and the aperiodic component measured with the Percept^TM^ neurostimulator between multiple recording moments (at least four weeks apart) in patients with PD who are chronically implanted with DBS electrodes. Furthermore, we aimed to determine whether movement and stimulation have a consistent effect on these features. The focus is on LFP signal features that are associated with motor deficits, particularly hypokinesia, caused by PD. Given the well-established modulatory effects of movement and stimulation on LFP beta power, and previous studies showing no time-related changes in beta based LFP features, we hypothesize that the effect of task execution and stimulation outweighs any temporal variability, and is consistent across time. In line with the E/I hypothesis^27^, dopaminergic medication and stimulation has shown to shift the system towards the prokinetic state, leading to more inhibition of the STN and thus an increase of the aperiodic exponent^30^. After withdrawal of dopaminergic medication, movement has led to a reduction of the aperiodic exponent in patients with PD^41^, suggestive of the pathological state. However, the consistency of the responsiveness of the aperiodic component to movement or stimulation across time has not yet been studied. Therefore, this aspect of the study was exploratory. Since the performance of an aDBS system strongly depends on the reliability of its feedback signal, understanding the (in)consistency of these spectral data features constitutes an important aspect for the development of aDBS algorithms for Parkinson’s disease.

## Results

**Fig. 1:**
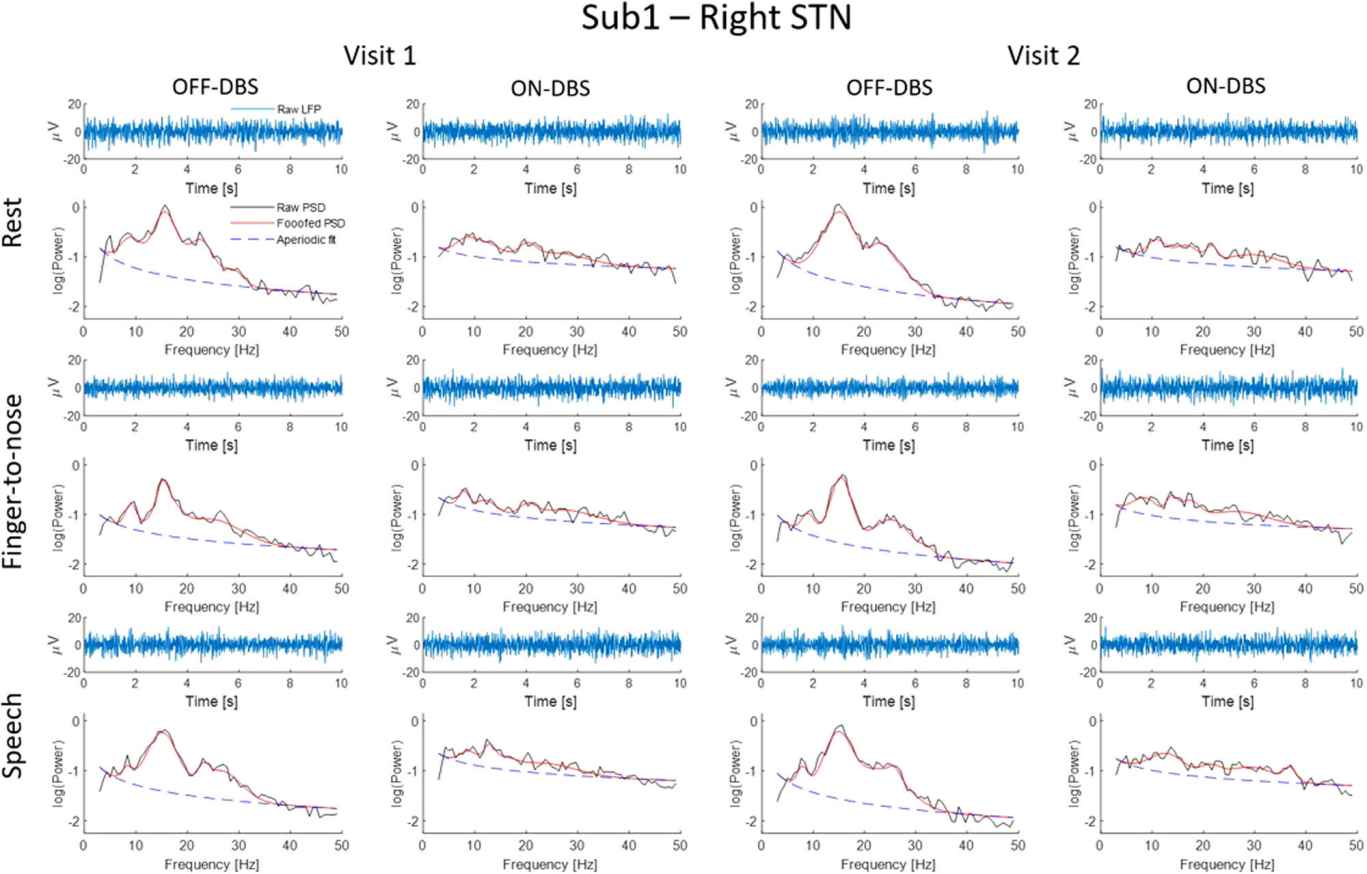
Local field potential (LFP) recordings in time- and frequency domain of the right subthalamic nucleus (STN) of subject 1 (Sub1). The blue line in the upper panels visualizes 10 s of the “raw” LFP signal in the time domain. The lower panels demonstrate the decomposition of the LFP recording into periodic and aperiodic signal components by means of the Fitting Oscillations & One Over F (FOOOF)^25^ algorithm: the “raw” power spectral density (PSD) (black line), FOOOF-fitted PSD (red line), and the aperiodic component of the PSD (dashed blue line).

Data from two visits of twelve participants (24 hemispheres) were used for the analyses (see Table 1 for patient details). The two visits had a mean inter-recording interval of 137 ± 78 days. For each visit, recordings with the Percept^TM^ neurostimulator were conducted twice: once with the stimulation switched off (OFF) and once with stimulation on (ON), while participants were on their regular anti-parkinsonian medication. LFP periodic and aperiodic spectral data features were extracted from the absolute power spectral densities and compared across visits, stimulation conditions and tasks. The tasks included resting, performing the finger-to-nose test with the left and right hand separately (movement), and reading aloud for 30 s (speech) (see Fig. 1 for example data from one participant and Fig. S1 in the Supplementary Materials for example data from all participants during one condition (Visit 1, ON-DBS)). The aperiodic component consists of an exponent and offset. The periodic component consists of beta peak width and power, and a brief analysis was performed for finely-tuned gamma at half the stimulation frequency.

**Table 1:** Patient characteristics

ID	Gender	Age [yrs] (mean ± SD)	DD [yrs] (mean ± SD)	Initial surgery [yrs] (mean ± SD)	Subtype	LEDD [mg] (median IQR)	ECG artifact [y/n]	Visit 1				Visit 2			
								Days after implantation (mean ± SD)	DBS settings L/R (contact(s)/amplitude/frequency/pulse width) [cathode(s)/mA/Hz/µs]		Beta peak selection L/R [Hz]	Days after visit 1 (mean ± SD)	DBS settings L/R (contact(s)/amplitude/frequency/pulse width) [cathode(s)/mA/Hz/µs]		Beta peak selection L/R [Hz]
1	M	73	20	8	T	499	Y	99	9-/2.1/180/90	2-/2.7/180/140	[15.1/15.7]	28	9-/2.1/180/90	2-/2.6/180/140	[15.0/15.3]
2	F	56	25	8	AR	560	Y	0	9-10-/1.6/80/60	2-/1.5/80/60	[22.0/31.9]	325	9-10-/1.3/130^a^/60	2-/1.4/130^a^/60	[22.2/30.9]
3	M	71	34	6	T	300	Y	50	9-/2.2/125^a^/60	1-/2.6/125^a^/60	[21.7/14.3]	94	9-/2.1/125^a^/60	1-/2.5/125^a^/60	[20.7/14.4]
4	F	52	18	6	AR	560	N	0	9-/2.3/125/60	1-/3.5/125/60	[18.8/21.6]	94	9-/2.3/130/60	1-/3.5/130/60	[18.6/19.5]
5	M	64	31	9	T	700	Y	50	9-/2.9/130/60	1-/4.0/130/60	[30.7/23.3]	120	9-/2.9/130/60	1-/4.0/130/60	[32.4/25.0]
6	M	69	13	7	T	450	Y	29	10-/2.8/180/60	2-/4.0/180/60	[22.4/19.4]	199	10-/2.6/180/60	2-/4.0/180/60	[20.0/17.4]
7	F	61	19	11	AR	1810	Y	24	9-/4.2/130/60	2-^a^/2.2/130/60	[23.2/17.8]	118	9-/4.2/130/60	2-^a^/2.1/130/60	[23.4/20.2]
8	M	68	10	5	AR	200	Y	67	10-^a^/1.9/130/60	1-/4.4/130/60	[30.5/26.1]	118	10-^a^/2.2/130/60	1-/4.6/130/60	[30.3/23.7]
9	M	76	16	7	AR	580	Y	87	10-/2.8/130/60	2-/2.8/130/60	[24.3/21.5]	224	10-/2.8/130/60	2-/2.8/130/60	[22.3/23.4]
10	F	58	16	11	T	400	Y	78	9-/2.6/130/60	1-2-/3.0/130/60	[14.8/17.6]	107	9-/2.6/130/60	1-2-/2.9/130/60	[15.5/18.7]
11	M	84	17	6	AR	100	Y	176	10-^a^/2.8/130/60	2-^a^/2.5/130/60	[20.2/22.0]	108	10-^a^/2.8/130/60	2-^a^/2.8/130/60	[21.6/21.5]
12	M	72	16	4	AR	1800	Y	46	9-/3.5/130/60	1-/3.7/130/60	[26.2/19.1]	104	9-/3.6/130/60	1-/3.8/130/60	[25.3/19.1]
	F: 4 M: 8	67 ± 9	20 ± 7	7 ± 2	AR: 7 T: 5	529.5 (325– 670)	Y: 11 N: 1	59 ± 49	2.6/133.3/62.5 ± 0.7/26.0/8.7	3.1/133.3/66.7 ± 0.9/26.0/23.1	22.5/20.9 ± 5.1/4.8	136.6 ± 77.6	2.6/137.9/62.5 ± 0.7/19.7/8.7	3.1/137.9/66.7 ± 0.9/19.7/23.1	22.3/20.8 ± 5.2/4.5

*DD* disease duration, *T* tremor, *AR* akinetic rigid.

^a^Suboptimal stimulation parameters for sensing purposes.

### Aperiodic component

**Fig. 2:**
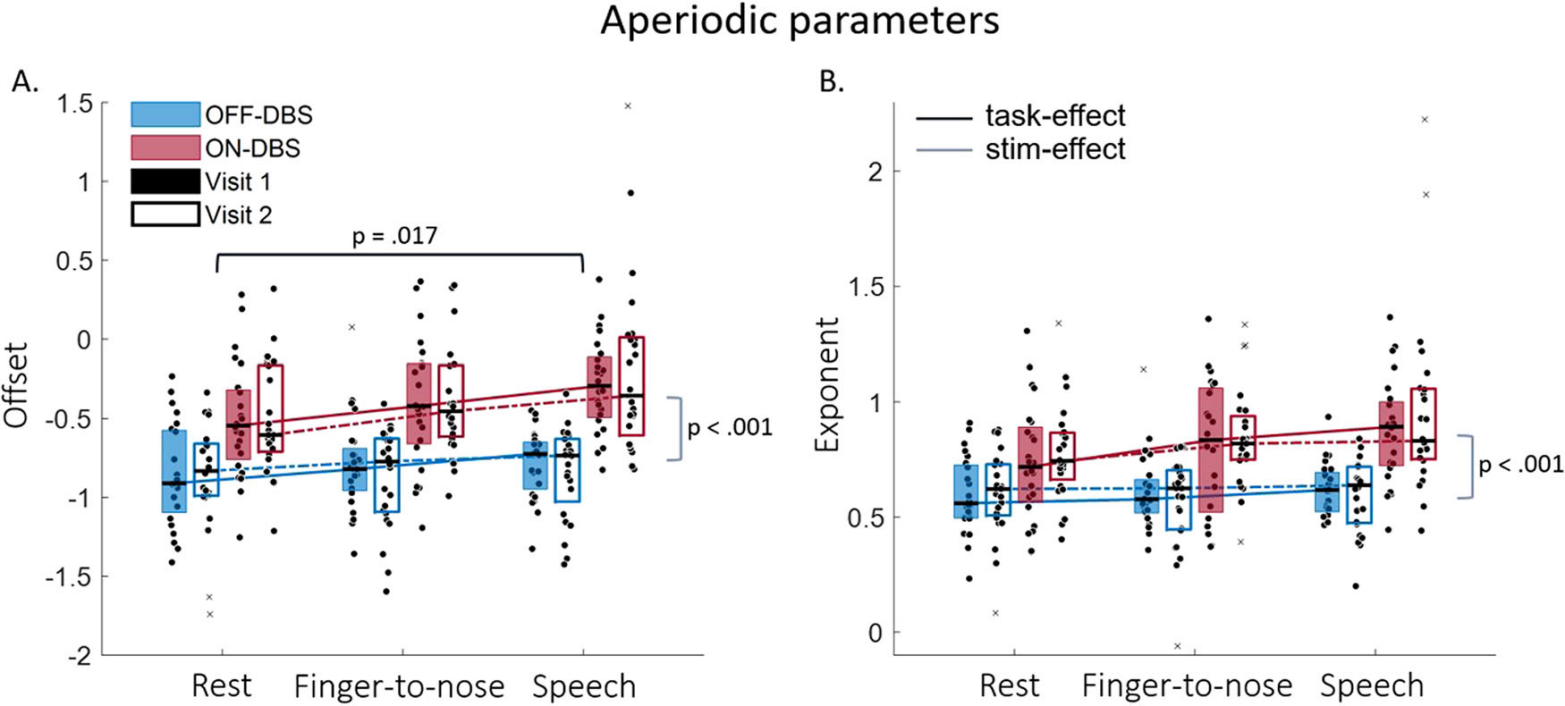
Median (Q1–Q3) aperiodic parameters for each visit, condition and task. The offset (**A**) and exponent (**B**) of the aperiodic component are categorized per task (rest, finger-to-nose, and speech). The color of the boxplot indicates stimulation condition OFF (blue) versus ON (red), a filled or empty boxplot indicates visit 1 (filled) versus visit 2 (empty). The solid (visit 1) and dashed (visit 2) lines highlight the interaction effects between Task and Stimulation. For both aperiodic parameters, a significant main effect of Task, main effect of Stimulation, and Task*Stimulation interaction effect was found. Significant post-hoc contrasts are indicated between tasks (black) and for the main effect of Stimulation (gray).

The outcomes for the aperiodic component are first described. Individual offset and exponent estimates per condition are visualized for each visit in Fig. 2. Group means and standard deviations are reported in Table S1 in Supplementary Materials.

For the aperiodic offset, the assumption of sphericity was not violated. The 2 × 2 × 3 one-way ANOVA showed a significant main effect of Task (*F*(2, 46) = 5.763, *p* = 0.006, *f* = 0.501) and Stimulation (*F*(1, 23) = 55.198, *p* < 0.001, *f* = 1.549) on the aperiodic offset. Post hoc analyses with Bonferroni correction revealed that the offset of the PSD was significantly increased during speech as compared to rest (*p* = 0.017), and after turning the stimulation ON as compared to OFF-DBS (*p* < 0.001). There was no significant main effect of Visit (*F*(1, 23) = 0.015, *p* = 0.903, *f* = 0.026), or interaction effect between Visit and Stimulation, between Visit and Task, or between Visit and Stimulation and Task, suggesting that estimated offset values remained comparable across visits. A statistically significant interaction effect was found between Task and Stimulation (*F*(2, 46) = 4.181, *p* = 0.021, *f* = 0.426), which means that the task-induced increase in offset was influenced by the stimulation condition (Fig. 2A). Specifically, paired samples T-tests showed that the offset increase during speech as compared to rest was larger during stimulation ON as compared to stimulation OFF (*t*(23) = 2.449, *p* = 0.022, *d* = 0.500). A trend towards a significantly larger offset increase during stimulation ON as compared to OFF was found for the finger-to-nose task as compared to rest (*t*(23) = 2.055, *p* = 0.051, *d* = 0.420).

Paired samples T-tests confirmed that the effect of stimulation on the offset was consistent over visits (*p* > 0.05 for the comparison of the difference between OFF- and ON-DBS over visits for all tasks). Similarly, the offset change between rest and the finger-to-nose task, as well as between rest and speech was consistent over visits for both OFF- as well as ON-DBS (*p* > 0.05). The difference in task-induced offset changes between stimulation OFF and ON was also consistent across visits for both the finger-to-nose task and speech (*p* > 0.05).

Given the main effects of Task and Stimulation, the Intraclass Correlation Coefficient (ICC) of the offset assessed over visits for all hemispheres was determined separately per task and stimulation condition, with values varying from poor to good reliability (ICC [95% confidence interval (CI)] OFF-rest moderate to good = 0.766 [0.528 0.892]; see Table 2 for a complete overview and section “Statistics” for the interpretation of ICC estimates). The ICC estimates for the task-induced and stimulation-induced increase of offset indicated poor to moderate reliability.

**Table 2:** Intraclass Correlation Coefficient (ICC) estimates and their 95% confidence intervals (CI) of the aperiodic offset and exponent assessed over visits for all hemispheres

Offset (ICC [95% CI])	OFF-DBS	Rest	0.766 [0.528 0.892]
		Finger-to-nose	0.581 [0.250 0.792]
		Speech	0.678 [0.392 0.845]
	ON-DBS	Rest	0.849 [0.682 0.932]
		Finger-to-nose	0.402 [−0.006 0.691]
		Speech	0.330 [−0.074 0.642]
	OFF-DBS	Rest to Finger-to-nose	0.282 [−0.105 0.603]
		Rest to Speech	0.282 [−0.128 0.610]
	ON-DBS	Rest to Finger-to-nose	0.215 [−0.215 0.568]
		Rest to Speech	0.470 [0.090 0.730]
	Δ OFF-ON-DBS	Rest	0.647 [0.333 0.831]
		Finger-to-nose	0.152 [−0.268 0.520]
		Speech	0.361 [−0.029 0.660]
		Rest to Finger-to-nose	-0.161 [−0.542 0.262]
		Rest to Speech	0.336 [−0.063 0.645]
Exponent (ICC [95% CI])	OFF-DBS	Rest	0.667 [0.363 0.841]
		Finger-to-nose	0.341 [−0.055 0.647]
		Speech	0.373 [−0.026 0.669]
	ON-DBS	Rest	0.752 [0.513 0.884]
		Finger-to-nose	0.277 [−0.143 0.609]
		Speech	0.361 [−0.031 0.660]
	OFF-DBS	Rest to Finger-to-nose	0.010 [−0.370 0.396]
		Rest to Speech	0.261 [−0.147 0.595]
	ON-DBS	Rest to Finger-to-nose	0.285 [−0.141 0.616]
		Rest to Speech	0.528 [0.166 0.764]
	Δ OFF-ON-DBS	Rest	0.757 [0.519 0.887]
		Finger-to-nose	0.289 [−0.112 0.612]
		Speech	0.319 [−0.072 0.631]
		Rest to Finger-to-nose	−0.023 [−0.421 0.379]
		Rest to Speech	0.440 [0.061 0.711]

ICC values are determined separately per task and stimulation condition, and also for task-induced and stimulation-induced changes. ICC estimates are interpreted to be poor (below 0.5), moderate (between 0.5 and 0.75), good (0.75 and 0.9), or excellent (0.9 or more).

For the aperiodic exponent, the assumption of sphericity was violated for Task (*χ*^2^(2) = 6.980, *p* = 0.030), and therefore a Greenhouse-Geisser correction was applied. The 2 × 2 × 3 one-way ANOVA showed a significant main effect of Task (*F*(1.572, 36.167) = 4.181, *p* = 0.031, *f* = 0.426) on the aperiodic exponent, where post hoc analysis with Bonferroni correction revealed no significant change during the finger-to-nose task as compared to rest (*p* = 0.553), but showed a trend towards a significant increase during speech as compared to rest (*p* = 0.053). The ANOVA also showed a significant main effect of stimulation (*F*(1, 23) = 26.447, *p* < 0.001, *f* = 1.072), where post hoc analyses revealed higher exponents while the stimulation was turned ON as compared to OFF (*p* < 0.001). No significant main effect of Visit was found (*F*(1, 23) = 0.281, *p* = 0.601, *f* = 0.111), or interaction effect between Visit and Stimulation, between Visit and Task, or between Visit and Stimulation and Task. Again, a significant interaction effect was found between Task and Stimulation (*F*(1.336, 30.734) = 4.352, *p* = 0.035, *f* = 0.435) (Fig. 2B). Paired samples T-test revealed a significant larger exponent increase during finger-to-nose as compared to rest during stimulation ON as compared to OFF (*t*(23) = 2.692, *p* = 0.013, *d* = 0.549), and a significant larger exponent increase during speech as compared to rest during stimulation ON as compared to OFF (*t*(23) = 2.551, *p* = 0.018, *d* = 0.521).

Paired samples T-tests confirmed that the effect of stimulation on the exponent was consistent over visits (*p* > 0.05 for the comparison of the difference between OFF- and ON-DBS over visits for all tasks). Similarly, the exponent change between rest and the finger-to-nose task, as well as between rest and speech was consistent over visits for both OFF- as well as ON-DBS (*p* > 0.05). The difference in task-induced exponent changes between stimulation OFF and ON was also consistent across visits for both the finger-to-nose task and speech (*p* > 0.05).

Again, the ICC of the exponent assessed over visits for all hemispheres was determined separately per task and stimulation condition (Table 2). ICC estimates varied from poor to moderate reliability, with a poor to good reliability level during OFF-rest (ICC [95% CI] = 0.667 [0.363 0.841]). The task-induced and stimulation-induced effects on the exponent showed poor to moderate reliability over visits (Table 2).

### Periodic component for all beta peaks

**Fig. 3:**
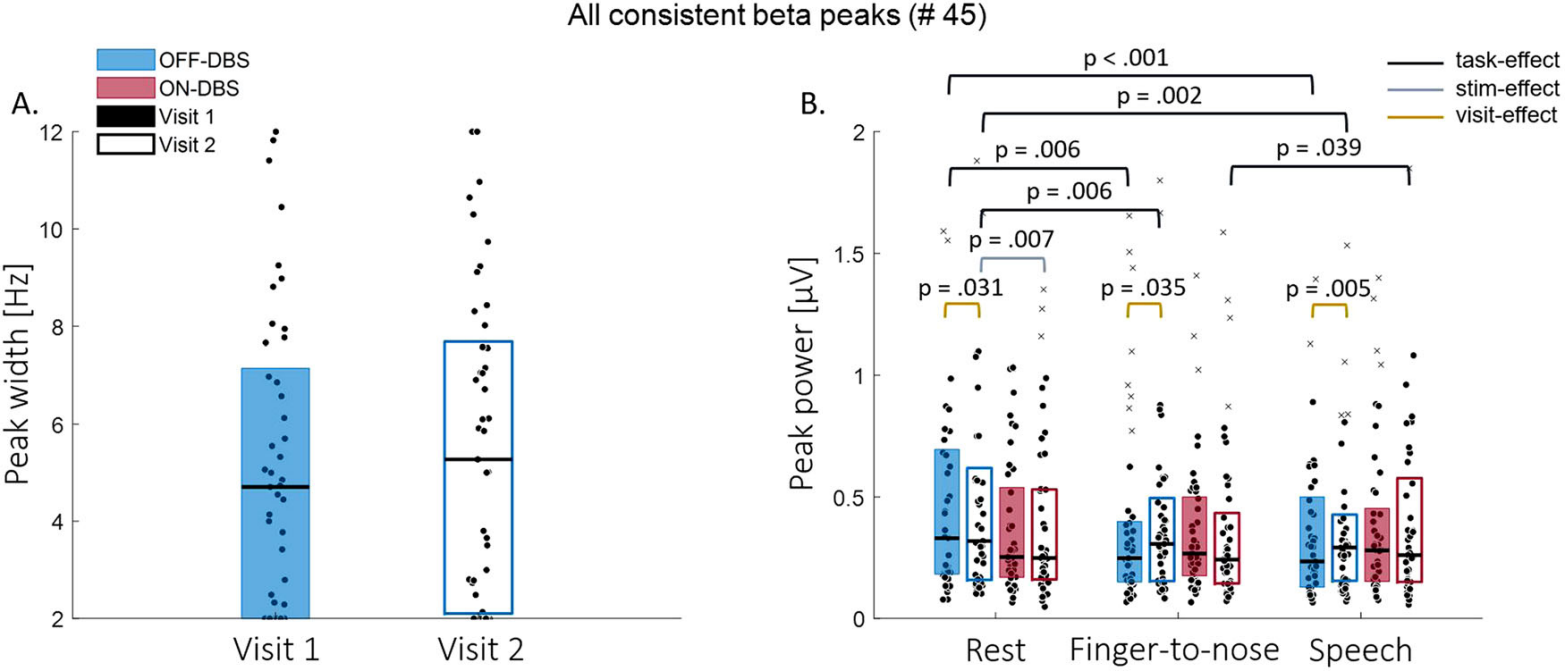
Median (Q1–Q3) periodic parameters of all consistent beta peaks. Beta peak width (**A**) is indicated for both visits. Beta peak power (**B**) is categorized per task (rest, finger-to-nose, and speech). The color of the boxplot indicates stimulation condition OFF (blue) versus ON (red), a filled or empty boxplot indicates visit 1 (filled) versus visit 2 (empty). Significant effects of task (black), stimulation (gray) and visit (dark yellow) are indicated.

In the recordings from all 24 hemispheres during visit 1 at rest with stimulation OFF, 63 peaks within the beta frequency range were found. In the OFF-rest recordings during visit 2, 64 beta peaks were found. Using a maximum between-visit deviation of 0.67 Hz between peak frequencies as a criterion for consistency revealed that recordings from 14 out of the 24 hemispheres (58%) contained one or more consistent peaks. If the maximum difference between peak frequencies was set at 2.5 Hz as criterion, consistent beta peaks were found in the recordings from all 24 hemispheres. Using this 2.5 Hz as criterion, 45 beta peaks were found to be consistent: for 8 hemispheres one consistent peak was found, for 11 hemispheres two consistent peaks and for 5 hemispheres three. The following results pertain to these 45 consistent beta peaks. Individual peak widths and power per condition are visualized for each visit in Fig. 3. Group medians and interquartile ranges are reported in Table S2 in the Supplementary Materials.

The width of the consistent beta peaks found during OFF-rest was not found to differ between the two visits (Fig. 3A; *Z* = 0.558, *p* = 0.577, *ɾ*_rb_ = 0.083). The corresponding ICC value (assessed over visits) was poor (ICC [95% CI] = 0.116 [−0.185 0.395]; Table 3).

**Table 3:** Intraclass Correlation Coefficient (ICC) estimates and their 95% confidence intervals (CI) of the beta peak width and power assessed over visits for all consistent peaks

Width all consistent peaks, *n* = 45 (ICC [95% CI])	OFF-DBS	Rest	0.116 [−0.185 0.395]
Power all consistent peaks, *n* = 45 (ICC [95% CI])	OFF-DBS	Rest	0.758 [0.597 0.860]
		Finger-to-nose	0.907 [0.837 0.948]
		Speech	0.889 [0.807 0.937]
	ON-DBS	Rest	0.854 [0.750 0.917]
		Finger-to-nose	0.834 [0.717 0.906]
		Speech	0.827 [0.707 0.901]
	OFF-DBS	Rest to Finger-to-nose	0.386 [0.115 0.606]
		Rest to Speech	0.118 [−0.173 0.392]
	ON-DBS	Rest to Finger-to-nose	0.350 [0.062 0.583]
		Rest to Speech	0.427 [0.158 0.637]
	Δ OFF-ON-DBS	Rest	0.730 [0.553 0.843]
		Finger-to-nose	0.682 [0.487 0.812]
		Speech	0.722 [0.545 0.837]
		Rest to Finger-to-nose	0.354 [0.077 0.582]
		Rest to Speech	0.112 [−0.177 0.387]

ICC values for power are determined separately per task and stimulation condition, and also for task-induced and stimulation-induced power changes. ICC estimates are interpreted to be poor (below 0.5), moderate (between 0.5 and 0.75), good (0.75 and 0.9), or excellent (0.9 or more).

The power (within a 4 Hz bandwidth) of the consistent beta peaks was significantly increased in visit 2 compared to visit 1 for all tasks while stimulation was OFF (Fig. 3B; rest: *Z* = 2.162, *p* = 0.031, *ɾ*_rb_ = 0.322; finger-to-nose: *Z* = 2.105, *p* = 0.035, *ɾ*_rb_ =0.314; speech: *Z* = 2.828, *p* = 0.005, *ɾ*_rb_ = 0.422). After turning the stimulation ON, the power of the consistent beta peaks was found to be similar in both visits while performing the different tasks (rest: *Z* = −0.164, *p* = 0.870, *ɾ*_rb_ = −0.024; finger-to-nose: *Z* = −0.897, *p* = 0.370, *ɾ*_rb_ = −0.134; speech: *Z* = 0.073, *p* = 0.942, *ɾ*_rb_ = 0.011). The ICC estimates of the power of the consistent beta peaks assessed over visits and separated per task and stimulation condition varied from good to excellent reliability (Table 3), indicating a high intra-peak consistency.

Compared to OFF-rest, the power at the frequency of the consistent beta peaks significantly decreased during the OFF-finger-to-nose task (visit 1: *Z* = −2.749, *p* = 0.006, *ɾ*_rb_ = −0.410; visit 2: *Z* = −2.760, *p* = 0.006, *ɾ*_rb_ = −0.411) and during OFF-speech (visit 1: *Z* = −3.708, *p* < 0.001, *ɾ*_rb_ = −0.553; visit 2: *Z* = −3.076, *p* = 0.002, *ɾ*_rb_ = −0.459). The power difference between rest and finger-to-nose, as well as between rest and speech in visit 1 was similar to the power differences observed during visit 2 (rest and finger to nose: *Z* = 0.175, *p* = 0.861, *ɾ*_rb_ = 0.026; rest and speech: *Z* = 0.130, *p* = 0.897, *ɾ*_rb_ = 0.019). However, ICC values of the power differences between rest and finger-to-nose (ICC [95% CI] = 0.386 [0.115 0.606]) and between rest and speech (ICC [95% CI] = 0.118 [−0.173 0.392]) indicated poor reliability (Table 3).

After turning the stimulation ON, no significant differences in beta power were found during finger-to-nose or speech compared to rest (*p* > 0.05 for the comparison between ON-rest and ON-finger-to-nose, and ON-rest and ON-speech in both visits). Only in visit 2, beta power was significantly increased during ON-speech as compared to ON-finger-to-nose (*Z* = 2.060, *p* = 0.039, *ɾ*_rb_ = 0.307). The power differences between rest and finger-to-nose, as well as between rest and speech were similar in both visits (rest and finger to nose: *Z* = −0.073, *p* = 0.942, *ɾ*_rb_ = −0.011; rest and speech: *Z* = 1.247, *p* = 0.212, *ɾ*_rb_ = 0.186). The ICC estimates of the power differences between rest and finger-to-nose (ICC [95% CI] = 0.350 [0.062 0.583] and between rest and speech (ICC [95% CI] = 0.427 [0.158 0.637]) indicated poor to moderate reliability (Table 3).

The power of the consistent beta peaks reduced after turning the stimulation ON during rest in visit 2 (*Z* = −2.703, *p* = 0.007, *ɾ*_rb_ = −0.403), but not in visit 1 (*Z* = −1.721, *p* = 0.085, *ɾ*_rb_ = −0.257). For the finger-to-nose and speech task, beta power remained the same after turning the stimulation ON in both visits (*p* > 0.05). The stimulation-induced power reduction during rest proved to be significantly larger during visit 2 as compared to visit 1 (Z = 2.658, *p* = 0.008, *ɾ*_rb_ = 0.396), but showed a moderate to good ICC estimate over visits (ICC [95% CI] = 0.730 [0.553 0.843]). Furthermore, the stimulation-induced power reduction during the finger-to-nose task (*Z* = 1.947, *p* = 0.052, *ɾ*_rb_ = 0.290) and speech (*Z* = 1.767, *p* = 0.077, *ɾ*_rb_ = 0.263) was found to be consistent over visits, although indicated a trend towards inconsistency. The ICC for the stimulation-induced power reduction during the finger-to-nose task (ICC [95% CI] = 0.682 [0.487 0.812]) and speech (ICC [95% CI] = 0.722 [0.545 0.837]) indicated moderate to good reliability (Table 3).

The difference in task-induced power changes between stimulation OFF and ON was consistent across visits (rest to finger-to-nose in OFF compared to ON: *Z* = 0.378, *p* = 0.705, *ɾ*_rb_ = 0.056; rest to speech in OFF compared to ON: *Z* = −0.006, *p* = 0.995, *ɾ*_rb_ = −0.001).

### Periodic component for selected beta peaks

**Fig. 4:**
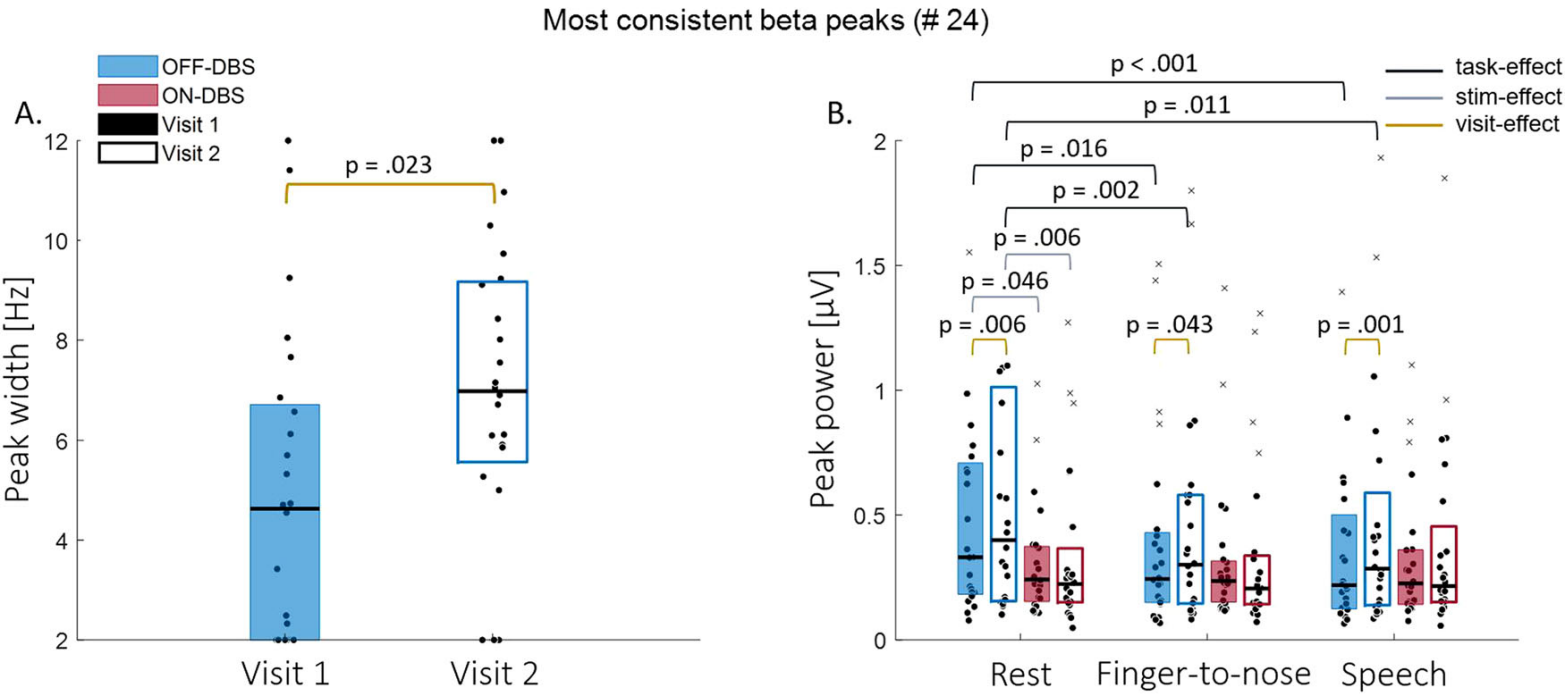
Median (Q1–Q3) periodic parameters of the most stimulation-responsive beta peaks. Beta peak width (**A**) is indicated for both visits. Beta peak power (**B**) is categorized per task (rest, finger-to-nose, and speech). The color of the boxplot indicates stimulation OFF (blue) or ON (red) condition, a filled or empty boxplot indicates visit 1 (filled) or visit 2 (empty). Significant effects of task (black), stimulation (gray) and visit (dark yellow) are indicated.

In 16 of the 24 hemispheres, multiple beta peaks were found to be consistent in the OFF-rest recordings of both visits. However, to implement aDBS in clinical practice, one peak needs to be selected as the frequency of interest. Of the consistent beta peaks found, determination of the most stimulation-responsive beta peak per hemisphere was based on the peak showing the largest stimulation-induced beta power reduction (expressed as the percentage deviation from “natural fluctuations”), averaged over both visits (please find a more detailed description in section “Data analysis”). A complete overview of this stimulation-induced power suppression of all consistent beta peaks can be found in the Supplementary Materials (Table S3). To mimic aDBS in clinical practice, the following results pertain to one beta peak per hemisphere (24 in total), where the most stimulation-responsive beta peak was selected in the 16 cases in whom multiple consistent beta peaks were found, and only one consistent beta peak was detected in the remaining 8 cases. Individual peak widths and power per condition are visualized for each visit in Fig. 4. Group medians and interquartile ranges are reported in Table S4 in the Supplementary Materials.

The width of the selected beta peaks found during OFF-rest was significantly different between the two visits (Fig. 4A; *Z* = 2.277, *p* = 0.023, *ɾ*_rb_ = 0.465). The corresponding ICC value (assessed over visits) was poor (ICC [95% CI] = 0.135 [−0.190 0.469]; Table 4).

**Table 4:** Intraclass Correlation Coefficient (ICC) estimates and their 95% confidence intervals (CI) of the beta peak width and power assessed over visits for the most stimulation-responsive peaks

Width stimulation-responsive peaks, *n* = 24 (ICC [95% CI])	OFF-DBS	Rest	0.135 [−0.190 0.469]
Power stimulation-responsive peaks, *n* = 24 (ICC [95% CI])	OFF-DBS	Rest	0.830 [0.622 0.925]
		Finger-to-nose	0.963 [0.896 0.985]
		Speech	0.864 [0.691 0.941]
	ON-DBS	Rest	0.848 [0.684 0.931]
		Finger-to-nose	0.935 [0.857 0.971]
		Speech	0.761 [0.529 0.889]
	OFF-DBS	Rest to Finger-to-nose	0.551 [0.211 0.775]
		Rest to Speech	0.416 [0.027 0.696]
	ON-DBS	Rest to Finger-to-nose	0.316 [−0.099 0.635]
		Rest to Speech	−0.193 [−0.571 0.234]
	Δ OFF-ON-DBS	Rest	0.805 [0.590 0.912]
		Finger-to-nose	0.898 [0.782 0.954]
		Speech	0.725 [0.466 0.870]
		Rest to Finger-to-nose	0.682 [397 0.828]
		Rest to Speech	0.217 [−0.198 0.565]

ICC values for power are determined separately per task and stimulation condition, and also for task-induced and stimulation-induced power changes. ICC estimates are interpreted to be poor (below 0.5), moderate (between 0.5 and 0.75), good (0.75 and 0.9), or excellent (0.9 or more).

Similar to the analyses with all 45 consistent beta peaks, when only analyzing the 24 selected peaks, there was a significant increase of beta power for all tasks with stimulation OFF in visit 2 as compared to visit 1 (rest: *Z* = 2.743, *p* = 0.006, *ɾ*_rb_ = 0.560; finger-to-nose: *Z* = 2.029, *p* = 0.043; *ɾ*_rb_ = 0.414; speech: *Z* = 3.229, *p* = 0.001, *ɾ*_rb_ = 0.659). With stimulation ON, no significant differences were found between the visits (rest: *Z* = 0.714, *p* = 0.475, *ɾ*_rb_ = 0.146; finger-to-nose: *Z* = 0.229, *p* = 0.819, *ɾ*_rb_ = 0.047; speech: *Z* = 0.629, *p* = 0.530, *ɾ*_rb_ = 0.128) (Fig. 4B). The ICC values of the power of the selected beta peaks (assessed over visits) separated per task and stimulation condition indicated good to excellent reliability (Table 4), reflecting a high intra-hemisphere consistency.

Similar to the findings for all 45 consistent peaks, the power of the selected beta peaks significantly decreased during the OFF-finger-to-nose task compared to OFF-rest (visit 1: *Z* = −2.400, *p* = 0.016, *ɾ*_rb_ = −0.490; visit 2: *Z* = −3.086, *p* = 0.002, *ɾ*_rb_ = −0.630) and during OFF-speech compared to OFF-rest (visit 1: *Z* = −3.429, *p* < 0.001, *ɾ*_rb_ = −0.700; visit 2: *Z* = −2.543, *p* = 0.011, *ɾ*_rb_ = −0.519). The power difference between rest and finger-to-nose, as well as between rest and speech in visit 1 was similar to these differences in power observed during visit 2 (rest and finger to nose: *Z* = -1.257, *p* = 0.209, *ɾ*_rb_ = −0.257; rest and speech: *Z* = −0.286, *p* = 0.775, *ɾ*_rb_ = −0.058). The ICC of the task-induced power changes between rest and finger-to-nose (ICC [95% CI] = 0.551 [0.211 0.775]) and between rest and speech (ICC [95% CI] = 0.416 [0.027 0.696]) during stimulation OFF assessed over visits were slightly higher as compared to the ICC estimates for all consistent peaks, though still indicating only poor to moderate reliability (Table 4).

After turning the stimulation ON, no significant differences in beta power were found during finger-to-nose or speech compared to rest (*p* > 0.05 for the comparison between ON-rest and ON-finger-to-nose, and ON-rest and ON-speech in both visits). Again, power differences between rest and finger-to-nose, as well as between rest and speech were similar in both visits (rest and finger to nose: *Z* = −0.029, *p* = 0.977, *ɾ*_rb_ = −0.006; rest and speech: *Z* = 1.000, *p* = 0.317, *ɾ*_rb_ = 0.204). The ICC of the task-induced power changes of the selected beta peaks between rest and finger-to-nose (ICC [95% CI] = 0.316 [−0.099 0.635]) and between rest and speech (ICC [95% CI] = −0.193 [−0.571 0.234]) during stimulation ON were slightly lower as compared to the ICC estimates for all consistent peaks and indicated poor reliability between visits (Table 4).

In contrast with the findings for all 45 consistent peaks, the power of the selected beta peaks reduced after turning the stimulation ON during rest in both visits (visit 1: *Z* = −2.000, *p* = 0.046, *ɾ*_rb_ = −0.408; visit 2: *Z* = −2.771, *p* = 0.006, *ɾ*_rb_ = −0.566). Again, beta power remained the same after turning the stimulation ON during the finger-to-nose and speech task in both visits (*p* > 0.05). The stimulation-induced power reduction was larger in visit 2 as compared to visit 1 during rest (*Z* = 2.143, *p* = 0.032, *ɾ*_rb_ = 0.437) and speech (*Z* = 2.143, *p* = 0.032, *ɾ*_rb_ = 0.437) but was found to be similar in both visits during the finger-to-nose task (*Z* = 1.314, *p* = 0.189, *ɾ*_rb_ = 0.268). The ICC estimates for the stimulation-induced power reduction varied from moderate (speech: ICC [95% CI] = 0.725 [0.466 0.870]) to good (rest: ICC [95% CI] = 0.805 [0.590 0.912]; finger-to-nose: ICC [95% CI] = 0.898 [0.782 0.954]) (Table 4), which were slightly higher compared to the findings for all consistent beta peaks (Table 3).

Similar to all consistent peaks, the difference in task-induced power changes of the 24 selected beta peaks between stimulation OFF and ON was consistent across visits (rest to finger-to-nose in OFF compared to ON: *Z* = 1.600, *p* = 0.110, *ɾ*_rb_ = 0.327; rest to speech in OFF compared to ON: *Z* = 0.114, *p* = 0.909, *ɾ*_rb_ = 0.023).

### Properties of the most stimulation-responsive beta peak

As the first OFF-rest recordings from 22 of the 24 hemispheres analyzed in this study contained multiple beta peaks, a number of follow-up analyses were performed to assist the decision which beta peak to best select “a priori’ as physiomarker for aDBS.

**Fig. 5:**
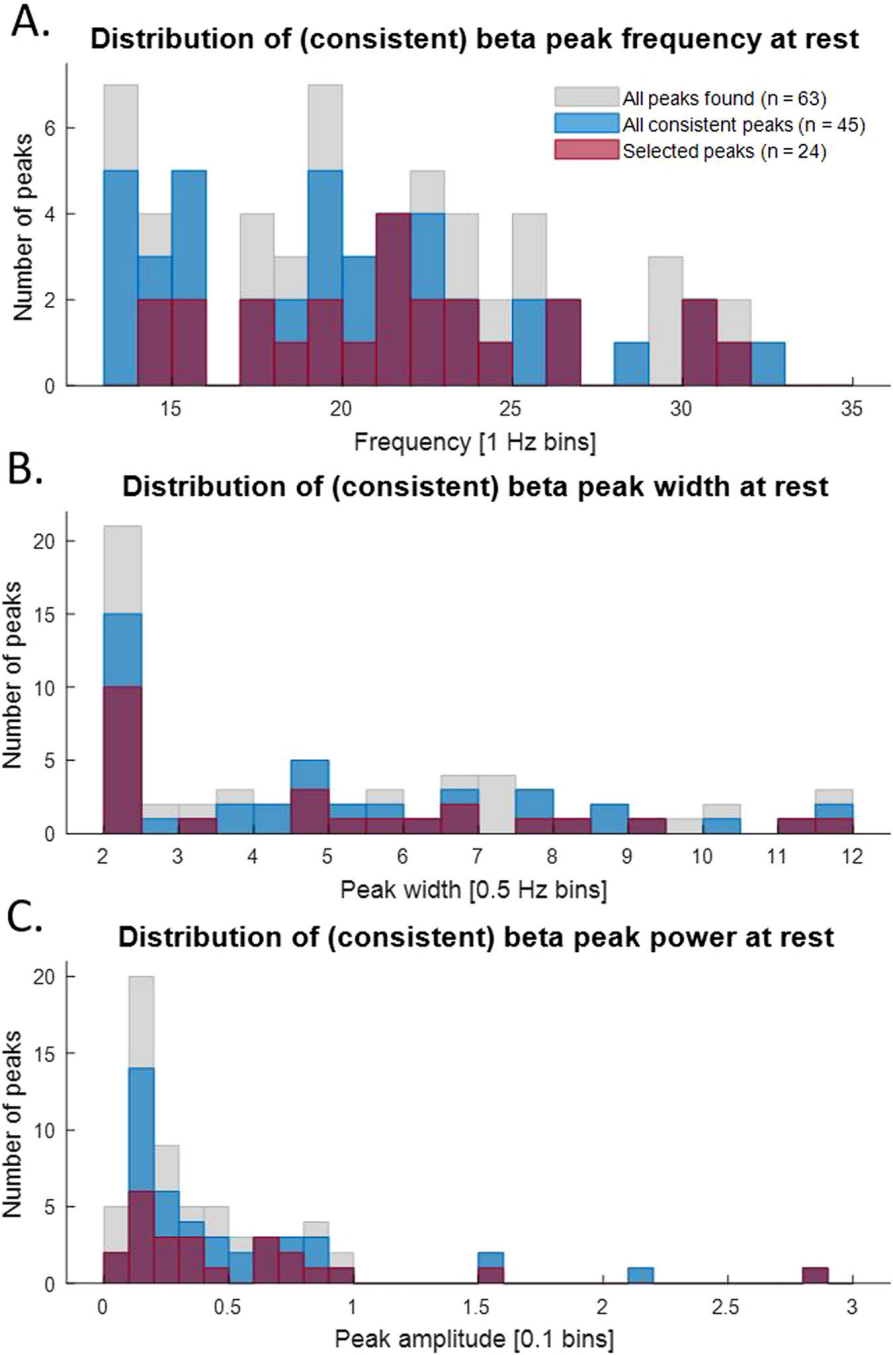
The distribution of beta peak frequency, width and power at rest. The distributions of peak frequency (**A**), peak width (**B**), and peak power (**C**) of all 63 peaks found in visit 1 (gray), the 45 consistent peaks (blue) and the 24 selected most stimulation-responsive peaks (red) are shown.

On average, the most stimulation-responsive beta peaks showed a beta power suppression during ON-rest of 211.5 ± 919.3% (mean ± standard deviation), where 100% corresponds with the natural fluctuations of beta peak power, defined as the absolute difference of beta power during OFF-rest between the two visits (please find a more detailed description in section “Data analysis”; see Supplementary Materials Table S3 for the complete overview of beta power suppression for all 45 consistent peaks). The frequency distribution of the selected beta peaks (Fig. 5A) resembled the distribution of all 63 peaks found during OFF-rest in visit 1. In 9 of the 22 hemispheres with multiple beta peaks during the first OFF-rest recording, the most stimulation-responsive beta peak was the peak with the highest frequency. In 5 hemispheres it was the peak with the lowest frequency, and in the other 8 hemispheres it was (one of) the middle peak(s). At the patient-level, the most stimulation-responsive beta peak was found at the same frequency for the left and right hemisphere in only 4 of the 12 participants (see Supplementary Materials Table S3).

The lower limit of peak width as fitted by FOOOF was set at 2 Hz, which is reflected in the distribution of estimated peak width values (Fig. 5B). Again, no clear distinction can be made between the most stimulation-responsive peaks as compared to all peaks detected during OFF-rest in visit 1. However, in 12 of the 22 hemispheres with multiple beta peaks during OFF-rest in visit 1, the peak with the smallest width was found to be the most stimulation-responsive beta peak. In 3 of these 11 cases, there were two beta peaks of 2 Hz width.

The power of the selected beta peaks also showed a similar distribution compared to that obtained for all detected peaks (Fig. 5C). In 9 of the 22 hemispheres with multiple beta peaks, the most stimulation-responsive beta peak appeared to be the peak with the highest power. In 14 of the 22 hemispheres, the peak selected as most stimulation-responsive appeared to be the peak demonstrating the largest beta suppression after turning the stimulation ON in visit 1 (which was part of the selection criteria). The peaks showing the largest beta suppression after turning the stimulation ON in the remaining 8 hemispheres were not found during OFF-rest in visit 2, and therefore not considered consistent (another part of the selection criteria).

### Presence of finely-tuned gamma at half the stimulation frequency

For the recordings of either or both of the visits from 12 of the 24 hemispheres, a Notch filter at half the stimulation frequency was applied, indicating the presence of stimulation-entrained finely-tuned gamma activity. Apart from a slight indication in the OFF-stimulation LFP recordings of the right hemisphere of Sub010 during both visits, finely-tuned gamma activity at half the stimulation frequency was only present during stimulation ON. In 7 of these 12 hemispheres, finely-tuned gamma activity at half the stimulation frequency was present during the ON-DBS condition in both visits.

## Discussion

The aim of this study was to establish and compare the long-term consistency of periodic (beta-based features) and aperiodic components derived from the PSD of STN-LFP recordings in patients with PD who are chronically implanted with DBS electrodes. Furthermore, the consistency of the effect of movement and stimulation on these LFP features was assessed. This evaluation was conducted to determine the potential of these signal features as physiomarkers for aDBS. LFP recordings were obtained at two time points at least 4 weeks apart. For the aperiodic offset and exponent, significant effects of Stimulation and Task execution were found. However, there was no significant main effect of Visit or interactions with the Visit factor. ICC values (range: [0.277 0.849]) indicated poor to moderate consistency. This demonstrated level of consistency of the (changes in the) aperiodic component is promising for future studies investigating the use of these signal features as reliable feedback markers for aDBS. However, notably higher consistency was found for beta power of the 45 consistent peaks that were detected, where ICC values during different tasks and stimulation conditions [0.761 0.963] indicated good to excellent reliability. Pairwise comparisons of beta peak power between visits 1 and 2 suggested lower consistency during stimulation OFF compared to ON. Interestingly, ICC estimates for task-induced power changes [−0.193 0.551] indicated only poor to moderate reliability, despite the absence of significant differences in task-induced power changes between visits. Contrary, stimulation-induced power changes significantly differed between visits, but showed moderate to good ICC estimates. Although outcomes were largely similar when selecting only the 24 beta peaks showing the strongest suppression response to stimulation, reliability of stimulation-induced power changes was slightly higher ([0.725 0.898] compared to [0.682 0.730]), and a significant stimulation-induced decrease in power during rest was found for both visits instead of only for visit 2. This suggests the possible importance of selecting optimal peaks for extracting reliable physiomarkers for aDBS.

This study adds to our understanding of the movement- and stimulation-related characteristics of the aperiodic component of STN-LFPs in patients with PD. Previous studies often removed the offset and exponent of the PSD to better analyze the periodic oscillations^42–47^. The clinical relevance of the STN-LFP aperiodic component, first explored by Hohlefeld et al.^26^, has recently gained renewed attention^30,31,41,48–50^ due to the introduction of new methods that separate PSD components, such as IRASA^51^ and FOOOF^25^. While some studies have reported no significant correlation between the aperiodic component and PD motor symptoms^50^, Belova et al.^41^ showed that the difference in the exponents between rest and voluntary movement significantly correlates with Movement Disorder Society–Unified Parkinson’s Disease Rating Scale Part III (MDS-UPDRS-III) scores. In contrast to Belova et al.^41^, who found a decrease of the exponent with the execution of voluntary movement, we observed the opposite pattern, with higher average exponent values during task execution, although post hoc comparisons with rest did not reach significance. These differences in outcomes could potentially be caused by differences in recordings performed intra-operatively versus in chronically implanted patients, and recordings performed after levodopa withdrawal versus on dopaminergic medication. Some previous studies have reported no significant effect of levodopa on the aperiodic component^48^, whereas others demonstrated dopaminergic medication to be accompanied with larger exponents^26,30^. Notably, Wiest et al.^30^ also reported an increase of the aperiodic exponent after turning stimulation ON, in line with the current study’s findings. The clinical relevance of the aperiodic component hence remains largely unclear due to the variability in data collection and signal processing protocols.

While it is important to understand how the aperiodic component relates to PD motor symptoms, dopaminergic medication, and DBS, its consistency over time is essential to determine its reliability as physiomarker for aDBS. To our knowledge, only one previous study analyzed the aperiodic component longitudinally^48^. Aside from an increase of the aperiodic component in the first 6 months post-implantation, the exponent and offset remained stable over the following year, consistent with the stability across visits observed in this study. The consistent increase in the aperiodic component following stimulation and task execution across visits shows potential for its use as a feedback marker for aDBS. However, ICC coefficients indicated less between-visit consistency for the aperiodic component as compared to beta peak power. This might be expected given the difficulties of estimating the exponent of the aperiodic component^52^, which is suggestive of introducing noise and, consequently, inconsistencies. In general, the decision of FOOOF parameter selection is data-specific and depends on the purpose of the analysis. Since the focus of the current study lies on determining LFP consistency it was decided to use the same frequency fitting range for all PSD analyses rather than performing an extensive investigation of the most suitable parameter choices per individual spectrum.

Regarding the periodic beta component, no significant differences in peak width were found for the 45 consistently present peaks at rest with stimulation OFF. However, ICC values for peak width showed poor reliability [0.116 0.135], potentially reflecting the skewed distribution in the FOOOF parameter range. The results of this study confirmed the known attenuation of beta band power following movement^11–13^ and stimulation^15–17^. Interestingly, beta peak power was found to decrease during speech, contrary to a recently reported increase during overt reading^21^ but in line with earlier studies that, though not investigating an overt reading task, reported reductions in beta power during speech-related activities^18–20^. In the current study, the task-induced reduction of beta power was only observed while the stimulation was OFF, and the stimulation-induced power reduction was only observed during rest. Additionally, the results indicate higher variability of beta peak power between visits during OFF-DBS. Practically, despite the fact that most power changes induced by task-execution or stimulation were found to be consistent over visits, the more variable beta power during DBS OFF might necessitate a periodic calibration—daily, weekly or monthly—or the use of advanced algorithms capable of dynamically adjusting thresholds for aDBS to account for variability and to accurately assess patient status.

Although the ICC estimates of the reduction of beta power after turning the stimulation ON during rest for only the most stimulation-responsive beta peaks indicated a slightly higher reliability as compared to all consistent beta peaks, the stimulation-induced power reduction remained inconsistent between the two visits. Determining the optimal beta peak that consistently responds to DBS activation on the basis of only one visit remains inconclusive based on the current results. Previous literature has suggested that beta peaks at lower frequencies (13–20 Hz) are more likely to be present in the pathological state^53^, whereas peaks at higher beta frequencies (20–35 Hz) are more likely to correlate with physiological motor circuit activity, associated with “healthy” movements^54^. Consequently, it was implied that low-frequency beta peaks should be selected for aDBS. Nonetheless, the current analyses revealed that both low and high-frequency beta peaks responded to task execution and stimulation. By contrast, a recent study found low-frequency beta peaks to respond to medication, while high-frequency beta peaks responded to stimulation^55^. Although recordings in the current study took place while participants were on medication, low-frequency beta peaks were still observed and showed a power reduction during stimulation. For the clinical application of aDBS based on the current study, it is recommended to perform multiple recording visits in order to determine the optimal beta peak(s) that consistently respond(s) to DBS in each individual patient.

Across studies, variable and generally low correlations between LFP physiomarkers and symptom severity have been reported^56^. To effectively differentiate between disease states or behaviors, it seems crucial to evaluate multiple (spectral) data features of the LFP signals. For example, the observed decrease in beta peak power during ON-DBS and task performance, despite an overall elevated PSD due to the increased aperiodic offset, indicates a distinct reduction in beta peak power unrelated to the noise floor or aperiodic offset. Beta power in the current study was determined using the original PSD, which included the aperiodic component; however, the contradictory effects of stimulation and task execution on periodic and aperiodic components suggest the necessity to decouple these signal features. This finding, consistent with Clark et al.^31^, suggests beta power and the aperiodic component as independent and uncorrelated signal features, potentially valuable for developing a multifaceted physiomarker to define clinical states^57^. Given the moderate consistency observed for the aperiodic component in this study (ICC values range: [0.277 0.849]), these features may be better suited as supplementary information on patients’ clinical state, rather than as a primary feedback signal. For example, cases where no beta peak is detected may be resolved by first extracting the aperiodic component. Alternatively, an elevated aperiodic component during movement with stimulation on could prevent the aDBS algorithm from detecting a movement-induced reduction of beta power, indicating that beta thresholds may need to be adjusted according to the detected level of the aperiodic component to more accurately reflect the clinical state.

Whereas LFP beta band features have been consistently linked to motor symptoms and levodopa responsiveness^6–10^, the latest developments in real-world aDBS suggest (cortical or STN) stimulation-entrained finely-tuned gamma activity as an alternative promising physiomarker^4^. Although Oehrn et al.^4^ analyzed finely-tuned gamma activity at half the stimulation frequency in all (six) STNs, only in half of the STNs it predicted high dopaminergic states and it was only used as a control signal for aDBS in one out of four participants. The strongest presence of finely-tuned gamma was seen in cortical signals. These findings correspond to the results of the current study, as finely-tuned gamma activity at half the stimulation frequency was only found in half of the STNs. Currently, finely-tuned gamma activity has been analyzed in a small group of patients, but further research is needed to assess whether these primary findings are generalizable to a larger patient cohort, thereby identifying under which circumstances finely-tuned gamma activity at half the stimulation frequency can be measured with DBS electrodes, i.e., specific patient characteristics, anatomical location of the DBS electrodes, patient dopaminergic or clinical state. This will also help identify patients in whom finely-tuned gamma activity cannot be utilized as physiomarker. In addition, physiomarkers might also be extracted from wearables or cortical (i.e., electrocorticography (ECoG)) recordings. Recent studies highlight the potential of extracting both periodic^58^ and aperiodic^59^ physiomarkers from ECoG recordings, and demonstrate the feasibility to be used as control signal for aDBS in a pilot study^4^. This, however, requires additional devices that pose an extra burden to the patient or surgical risks, though preserves the option to select all contacts on the DBS electrode for therapy optimization.

A number of methodological aspects of the current study deserve further discussion. First of all, the fact that participants were still on their regular medication schedule, which has repeatedly proven to influence beta band power, poses a limitation. The effect of dopaminergic medication on the aperiodic component is not evaluated in this study, and contradictory results of previous studies remain unclear^30,48^. Although the daily dose of dopaminergic medication generally reduces drastically in patients that are treated with DBS, the majority of patients still require some medication. Consequently, in case of aDBS, patients will remain in a fluctuating medication state while stimulation levels vary. Thus, LFP fluctuations observed in the current study are representative of how LFPs are likely to vary in clinical practice. Further research involving repeated LFP recordings in participants withdrawn from dopaminergic medication will allow to determine whether medication withdrawal is the critical determinant potentially enabling reliable selection of the optimal consistent stimulation-responsive beta peak based on a single visit for clinical application of aDBS.

Secondly, given the published^60–62^ and practically experienced problems with artifacts measured with the Percept neurostimulator, especially in patients implanted with legacy (3389) electrode leads as in our cohort, which are more susceptible to signal contamination compared to the newer SenSight leads specifically designed for LFP sensing^61,63^, we are aware of potential artifacts contaminating the LFP recordings. We mitigate these by using the same recording mode during the OFF- and ON-DBS conditions, visually inspecting all recordings, and applying extensive cleaning if needed. In doing so, we have ruled out as much as possible the possibility that the reported task-induced effects on the LFP features are due to movement, ECG, or stimulation artifacts. However, it is impossible to entirely rule out the possibility that our findings are still confounded by remaining artifacts that are not directly apparent in the time series or power spectral densities.

Thirdly, although previous studies investigating the spectral data features of STN-LFPs of patients with PD often included similar number of participants^55,56,64^, the rather small sample size with only two repetitions of relatively short duration of measurement poses a limitation of the current study. Luckily, the emergence of fully implanted bidirectional neurostimulators enables further investigation of physiomarker consistency with larger cohorts and more frequent measurement sessions. The present study should be viewed as a validation of the feasibility to detect these features and as guidance on aspects to consider in the ongoing development and clinical implementation of aDBS systems. Future research will need to address whether the observed changes of these physiomarkers indeed reflect alterations in symptom severity or are solely attributable to stimulation effects or motor task execution by incorporating clinical measures, such as UPDRS-III scores, to assess the stability and reliability of LFP signal changes in relation to PD symptom dynamics. Furthermore, whether these physiomarkers indeed also lead to successful aDBS applications remains to be determined.

Finally, the current study considered the LFP physiomarkers to be consistent if no significant effect of the factor visit was found. While the presence of a significant effect can be taken as evidence for inconsistency, the lack of a significant effect is merely an indication of possible consistency. A lack of group-level effects might go hand-in-hand with consistent within-subject changes that differ in sign between subjects. Therefore, ICC values for both periodic and aperiodic components were included. Alternatively, Bayesian methods provide an estimate of the probability of rejecting the null hypothesis, but also offer an estimate of the credibility of the alternative hypothesis, allowing for a more nuanced understanding of the data^65,66^. However, Bayesian approaches require the selection of a prior, which introduces an additional layer of complexity. In small datasets such as the one used in this study, the choice of prior can significantly influence the results (known as prior sensitivity)^67^. This issue can be mitigated by selecting a non-informative prior, but this may lead to wide credible intervals and increased uncertainty, thus limiting the ability of Bayesian methods to provide clarity in the current study. Given the increased complexity and computational intensity of Bayesian analysis, it was ultimately not pursued in this study. It should further be noted that ICC values were determined for all peaks and/or hemispheres as independent observations. This method overlooks within-patient variability, which may result in an overestimation of consistency^68^.

To conclude, in order for aperiodic or periodic components of STN-LFP signals to be useful as physiomarkers for aDBS in PD, these signal features need to be consistent markers of patients’ behavioral and/or clinical state. This study proves the moderate between-visit consistency of the aperiodic offset and exponent, but good to excellent consistency of beta peak power over the course of several weeks to months. Despite the poor to moderate ICC values, task-induced aperiodic and power changes were statistically comparable across visits. Stimulation-induced changes were only consistent for the aperiodic components. The results remained inconclusive regarding the peak frequency of the most stimulation-responsive peak in case multiple peaks within the beta range were observed in the OFF stimulation recording. Given the variable nature of beta peak power and the demonstrated inability to firmly select the most stimulation-responsive beta peak on the basis of only one visit, the clinical applicability of aDBS will most likely require multiple recording visits and periodic calibration.

## Methods

### Participants

Twelve patients with Parkinson’s disease were included in this study (see Table 1 for patient details). All patients were bilaterally implanted in the subthalamic nucleus (STN) between 2011–2019 (electrode lead model 3389, Medtronic, Minneapolis, MN). Due to battery depletion, the pulse generator was replaced with the Percept^TM^ PC neurostimulator (Medtronic, Minneapolis, USA) in the context of standard clinical care between January 2021 and March 2023. Local field potential recordings were performed in all participants within one year after replacement surgery while participants were on their regular anti-parkinsonian medication. Five participants came to the hospital twice to participate in the study. Seven participants made an additional third visit. However, due to different causes—synchronization problems (subject 1 visit 1), inconsistency in the contact pair used for stimulation (subject 2 visit 2), missing LFP data from the right hemisphere (subject 6 visit 2), problems with the external recording system (subject 8 visit 1), and missing LFP data during the finger-to-nose task due to (movement) artifacts (subject 9 visit 2)—complete data sets for all three visits were only available for two participants (subjects 5 and 7). Consequently, we decided to only include two visits in further analyses.

Patients’ disease duration was on average 20 ± 7 years and their time from lead implantation was 7 ± 2 years (see Table 1 for patient details). Median levodopa-equivalent dose (LEDD) at the time of the first visit was 530 mg (IQR 325–670). Participants had their first recording on average 59 ± 46 days after Percept^TM^ implantation. Two participants had their first recording on the day of battery replacement. This study was approved by the local ethics committee (Medisch Ethische Toetsingscommissie Amsterdam Medical Center (AMC)) and was carried out in accordance with the Declaration of Helsinki (NL74645.018.20). Informed written consent was received from all patients.

### Data acquisition

For each visit, bilateral STN-LFP recordings were obtained. For each visit, LFP recordings during resting state and a series of movement tasks using the BrainSense^TM^ Streaming feature of the Percept^TM^ neurostimulator^69–71^ once with stimulation switched on at 0 mA (OFF) and once on stimulation (ON) with the patients’ clinically effective stimulation parameters (Table 1). Leaving the neurostimulator technically “active” or “on” but at 0 mA while recording the stimulation OFF condition, instead of technically turning the stimulator off, avoids confounded differences in LFP features as a result of scaling differences caused by different recording modes^60,62,72^. The two contact points surrounding the monopolar contact selected for clinical stimulation were used for bipolar recordings. As the LFP recordings require a symmetric dipole (“sandwiching”) around the stimulation electrode contact point, in case clinically effective stimulation was set at the ventral or dorsal electrode contact, the adjacent contact was activated during this study (Table 1). Eight participants performed the recordings first with stimulation OFF and then ON, the other four participants first with stimulation ON and then OFF. Regardless of the order of conditions, there was a 15-min break before the recording with stimulation OFF was conducted to allow for proper wash-out of stimulation effects^73^. Participants lay on a bed with the headrest in 45°, or sat comfortably in a chair with the arms supported, and were instructed not to talk or sleep during recordings. LFP signals were recorded at a sampling frequency of 250 Hz for ~60 s while the participants were at rest, for 30 s while participants performed the finger-to-nose tremor test (for both left and right hand separately) and while reading aloud for 30 s. Although not further analyzed in the current study, participants also performed several other tests that are part of the MDS-UPDRS-III assessment, including finger tapping, pro- and supination movements of the hands, and postural hold. Since the aim of the current study was not to conduct a comprehensive analysis of all UPDRS-III tasks, but rather to investigate the stability and reliability of the LFP signals across different states over time, the three selected tasks—rest, finger-to-nose, and speech—were chosen to contrast a baseline state with two distinct motor tasks representing hypokinesia in Parkinson’s disease. Including speech alongside the finger-to-nose task allows the assessment of the stability and reliability of LFP features across diverse motor behaviors that are not only biomechanically distinct but also critical for daily life. A simultaneous bipolar ECG signal was recorded using two electrodes placed on the right and left shoulder (locations: RA and LA of ECG 12 Lead Placement; TMSi Porti amplifier: monopolar, average reference, anti-aliasing low-pass filter with a cut-off frequency of 500 Hz and sampling frequency of 2048 Hz, TMSi, The Netherlands). LFP and ECG signals were synchronized by slightly ramping up and down the stimulation amplitude, as previously described^62,71,74^. The ECG signals were subsequently downsampled to 250 Hz to match the sampling rate of the LFP signals. Two tri-axial accelerometers (±3 g, TMSi, The Netherlands) were attached to the distal phalanx of the middle finger of each hand, though not further analyzed in this study. Event markers were co-recorded in the ECG acquisition software to indicate the beginning and ending of each task.

### Data analysis

The raw, continuous LFP signals were cleaned by applying each of the following filters consecutively: a) A 3-Hz high-pass filter (bidirectional 4th order Butterworth), b) A Hampel filter for the frequency range of 30–125 Hz (using a recording-specific threshold (default at 3.0 but varying between 2.6 and 3.5) to remove prominent narrow-band peaks at recurrent frequencies)^75^, c) Notch filters for removing line noise at 50 Hz and narrow-band artifacts at other frequencies if needed (typically at 65, 79.85, 80, 90.5, 120, and/or 125 Hz). The presence of finely-tuned gamma activity was determined based on whether a Notch filter had been applied at half the stimulation frequency, d) The simultaneously recorded ECG signal was used for detecting and suppressing ECG artifacts, if needed, by means of singular value decomposition (SVD) using recording-specific components (generated from 250 ms before until 400 ms after each (manually reviewed) detected R-peak) containing typical ECG peaks of the QRS-complex that outweigh potential oscillations outside this region^62^, e) A Hampel filter for artifacts within the frequency range 3.8–40 Hz (using a recording-specific threshold, default at 3.0 but varying between 2.6 and 3.3) that became apparent after ECG cleaning. The event markers were used to select the time period of task performance from the cleaned LFP signals. Analyses of the finger-to-nose tremor test were only performed for LFP recordings from the contralateral hemisphere. The LFP signals from both hemispheres were analyzed during rest and the speech task. Some of the task-specific LFP signals had remaining (e.g., ECG or movement) artifacts. For consistent comparisons, two 10-s segments of each task-specific LFP signal containing samples only within the boundaries of its mean ± 5 standard deviations were selected. For 9 out of 288 task-specific LFP recordings only one of such 10-s artifact-free segments could be selected.

Spectral analyses were performed on LFP data using Fieldtrip software^76^ for MATLAB (version R2023b, MathWorks, Natick, MA) and the FOOOF software package (version 1.1.0, https://github.com/fooof-tools/fooof) for Python^25^. All 10-s segmented LFP signals were analyzed using Welch’s method (MATLAB function *pwelch*) with 50% overlapping Hamming windowed segments of 1.5-s to obtain a power spectral density (PSD) at a 0.67 Hz frequency resolution for each resting state period, movement task and speech period. The average PSD of the two 10-s sections of each task were taken except for the 9 recordings for which only one 10-s section could be used due to artifacts. The (average) absolute PSD was decomposed into periodic and aperiodic signal components by the FOOOF algorithm for each hemisphere-, visit-, task- and condition-specific LFP recording. The exponent and the offset of the aperiodic component of the PSD were determined across the frequency range 3–49 Hz by fitting a fixed aperiodic mode with no knee and maximally 4 Gaussian fits (to capture spectral peaks) of minimally 2 and maximally 12 Hz width. This frequency fitting range was selected to encompass the full beta frequency range allowing accurate fitting of beta peaks, while also avoiding spectral dips due to signal cleaning, spectral flattening, and periodic components crossing the fitting range borders^52^. Subsequently, the fitted parameter values for peak frequency, power and width of all peaks within the 13–35 Hz frequency range (beta peaks) were extracted.

The Percept^TM^ neurostimulator uses a 5 Hz bandwidth surrounding the selected frequency (selected frequency ±2.5 Hz) to calculate beta power during at home recordings. For translation to clinical practice, beta peaks found during rest with stimulation OFF (OFF-rest) that had a peak frequency difference of maximum 2.5 Hz between visits were defined as consistent. Based on prior research, beta peak power is anticipated to be highest during the OFF-rest recordings, which was therefore taken as a reference condition. Due to the frequency resolution of 0.67 Hz used to obtain the PSDs, the current study used a 4 Hz bandwidth (unlike the 5 Hz used by the Percept^TM^ neurostimulator) to calculate beta peak power (using MATLAB function *trapz*), thereby capturing an equal number of frequency bins on either side of the selected frequency. Per hemisphere, the frequency range as determined based on the OFF-rest recordings was used to calculate the beta peak power for all other recordings.

To implement aDBS in clinical practice, per hemisphere one peak needs to be selected as the frequency of interest. Therefore, in case of multiple consistent beta peaks, determination of the most stimulation-responsive beta peak per hemisphere was based on the peak showing the largest suppression of beta power after turning the stimulation ON, averaged over both visits. Beta power suppression was expressed as the percentage deviation from “natural fluctuations’, which was defined as the absolute difference of beta power during rest and stimulation OFF between two visits. The distribution of the frequency, width and power of the most stimulation-responsive beta peaks as compared to all (consistent) beta peaks was used to explore the possibility of deciding which beta peak to best select “a priori” as physiomarker for aDBS.

### Statistics

Statistical analyses were performed using IBM SPSS Statistics (version 28.0). Shapiro-Wilk tests were performed to determine normality of the aperiodic (offset and exponent) as well as periodic (beta peak power and width) components.

The consistency of the aperiodic offset and exponent between visits and conditions were statistically tested using a 2 × 2 × 3 one-way ANOVA with repeated measures (Visits × Stimulation condition × Tasks) or Friedman test, where appropriate. A Greenhouse-Geisser correction was used for the repeated measures ANOVA if Mauchly’s Test of Sphericity indicated that the assumption of sphericity had been violated. Pairwise comparisons with a Bonferroni correction were conducted for post hoc analysis of significant effects, where Bonferroni-corrected *p*-values were reported. In case a significant task-induced, stimulation-induced or interaction effect was detected, the consistency of these effects was tested across visits and conditions by means of paired samples T-tests or Wilcoxon signed-rank tests, where appropriate. In addition, Intraclass Correlation Coefficient (ICC) estimates and their 95% confidence intervals (CI) based on a two-way mixed effects analysis of variance, using single measurement and an absolute agreement definition, were calculated. ICC estimates were assessed over visits for all hemispheres and determined separately per task and stimulation condition. ICC values were interpreted to be poor (below 0.5), moderate (between 0.5 and 0.75), good (0.75 and 0.9), or excellent (0.9 or more)^77^.

The consistency of the periodic (beta peak power and width) component between visits, tasks and stimulation conditions of all consistent beta peaks, as well as of the most stimulation-responsive beta peaks, was statistically tested by means of Wilcoxon signed-rank tests and ICC estimates with 95% CI based on a two-way mixed effects analysis of variance (using single measurement and an absolute agreement definition). The consistency of task-induced and stimulation-induced effects on the periodic component was assessed across visits using Wilcoxon signed-rank tests and ICC estimates. Other than reporting on the number of (consistent) beta peaks (per hemisphere), no further statistical testing was performed on beta peak frequency. LFP physiomarkers were considered consistent if no significant main effect (*p* > 0.05 after adjustment, if applicable) of Visit or interaction effect with Visit was found for the ANOVA or Wilcoxon tests. Effect sizes were calculated as Cohen’s *f* for the one-way ANOVAs with repeated measures (“*f*”), Cohen’s *d* for the paired-samples T-tests (“*d*”), and rank-biserial correlation coefficient for Wilcoxon signed-rank tests (“*r*_rb_”).

## Supplementary information

LFP_Consistency_Supplementary Materials_Revised

## Data Availability

Depending on the type of data and associated privacy regulations, data from the current study can be made available via the corresponding author, upon reasonable request.

## Code availability

The code used to analyze data during the current study will not be made publicly available, but is available from the corresponding author on request.

## References

[1] Deuschl, G. et al. European academy of neurology/Movement Disorder Society-European section guideline on the treatment of Parkinson’s disease: I. Invasive therapies. *Eur. J. Neurol.* **29**, 2580–2595 (2022). 35791766 10.1111/ene.15386

[2] Limousin, P. et al. Effect on parkinsonian signs and symptoms of bilateral subthalamic nucleus stimulation. *Lancet* **345**, 91–95 (1995). 7815888 10.1016/s0140-6736(95)90062-4

[3] Little, S. et al. Bilateral adaptive deep brain stimulation is effective in Parkinson’s disease. *J. Neurol. Neurosurg. Psychiatry* **87**, 717–721 (2016). 26424898 10.1136/jnnp-2015-310972 PMC4941128

[4] Oehrn, C. R. et al. Chronic adaptive deep brain stimulation versus conventional stimulation in Parkinson’s disease: a blinded randomized feasibility trial. *Nat. Med.*10.1038/s41591-024-03196-z (2024). 39160351 10.1038/s41591-024-03196-z PMC11826929

[5] Swann, N. C. et al. Adaptive deep brain stimulation for Parkinson’s disease using motor cortex sensing. *J. Neural Eng.* **15**, 046006 (2018). 29741160 10.1088/1741-2552/aabc9b PMC6021210

[6] Alonso-Frech, F. et al. Slow oscillatory activity and levodopa-induced dyskinesias in Parkinson’s disease. *Brain* **129**, 1748–1757 (2006). 16684788 10.1093/brain/awl103

[7] Kühn, A. A., Kupsch, A., Schneider, G. H. & Brown, P. Reduction in subthalamic 8-35 Hz oscillatory activity correlates with clinical improvement in Parkinson’s disease. *Eur. J. Neurosci.* **23**, 1956–1960 (2006). 16623853 10.1111/j.1460-9568.2006.04717.x

[8] Kühn, A. A. et al. Pathological synchronisation in the subthalamic nucleus of patients with Parkinson’s disease relates to both bradykinesia and rigidity. *Exp. Neurol.* **215**, 380–387 (2009). 19070616 10.1016/j.expneurol.2008.11.008

[9] Ray, N. J. et al. Local field potential beta activity in the subthalamic nucleus of patients with Parkinson’s disease is associated with improvements in bradykinesia after dopamine and deep brain stimulation. *Exp. Neurol.* **213**, 108–113 (2008). 18619592 10.1016/j.expneurol.2008.05.008

[10] Weinberger, M. et al. Beta oscillatory activity in the subthalamic nucleus and its relation to dopaminergic response in Parkinson’s disease. *J. Neurophysiol.* **96**, 3248–3256 (2006). 17005611 10.1152/jn.00697.2006

[11] Cassidy, M. et al. Movement-related changes in synchronization in the human basal ganglia. *Brain* **125**, 1235–1246 (2002). 12023312 10.1093/brain/awf135

[12] Kuhn, A. A. et al. Event-related beta desynchronization in human subthalamic nucleus correlates with motor performance. *Brain* **127**, 735–746 (2004). 14960502 10.1093/brain/awh106

[13] Levy, R. et al. Dependence of subthalamic nucleus oscillations on movement and dopamine in Parkinson’s disease. *Brain* **125**, 1196–1209 (2002). 12023310 10.1093/brain/awf128

[14] Tinkhauser, G. et al. Electrophysiological differences between upper and lower limb movements in the human subthalamic nucleus. *Clin. Neurophysiol.* **130**, 727–738 (2019). 30903826 10.1016/j.clinph.2019.02.011 PMC6487671

[15] Bronte-Stewart, H. et al. The STN beta-band profile in Parkinson’s disease is stationary and shows prolonged attenuation after deep brain stimulation. *Exp. Neurol.* **215**, 20–28 (2009). 18929561 10.1016/j.expneurol.2008.09.008

[16] Eusebio, A. et al. Deep brain stimulation can suppress pathological synchronisation in parkinsonian patients. *J. Neurol. Neurosurg. Psychiatry* **82**, 569–573 (2011). 20935326 10.1136/jnnp.2010.217489 PMC3072048

[17] Kühn, A. A. et al. High-frequency stimulation of the subthalamic nucleus suppresses oscillatory β activity in patients with Parkinson’s disease in parallel with improvement in motor performance. *J. Neurosci.* **28**, 6165–6173 (2008). 18550758 10.1523/JNEUROSCI.0282-08.2008 PMC6670522

[18] Anzak, A. et al. A gamma band specific role of the subthalamic nucleus in switching during verbal fluency tasks in Parkinson’s disease. *Exp. Neurol.* **232**, 136–142 (2011). 21872587 10.1016/j.expneurol.2011.07.010

[19] Chrabaszcz, A. et al. Subthalamic nucleus and sensorimotor cortex activity during speech production. *J. Neurosci.* **39**, 2698–2708 (2019). 30700532 10.1523/JNEUROSCI.2842-18.2019 PMC6445998

[20] Hebb, A. O., Darvas, F. & Miller, K. J. Transient and state modulation of beta power in human subthalamic nucleus during speech production and finger movement. *Neuroscience* **202**, 218–233 (2012). 22173017 10.1016/j.neuroscience.2011.11.072 PMC3286522

[21] Avantaggiato, F. et al. Intelligibility of speech in Parkinson’s disease relies on anatomically segregated subthalamic beta oscillations. *Neurobiol. Dis.* **185**, 106239 (2023). 37499882 10.1016/j.nbd.2023.106239

[22] Swann, N. C. et al. Gamma oscillations in the hyperkinetic state detected with chronic human brain recordings in parkinson’s disease. *J. Neurosci.* **36**, 6445–6458 (2016). 27307233 10.1523/JNEUROSCI.1128-16.2016 PMC5015781

[23] Ricciardi, L., Apps, M. & Little, S. Uncovering the neurophysiology of mood, motivation and behavioral symptoms in Parkinson’s disease through intracranial recordings. *npj Parkinson’s Dis.* **9**, 136 (2023). 37735477 10.1038/s41531-023-00567-0 PMC10514046

[24] Miller, K. J., Sorensen, L. B., Ojemann, J. G. & Den Nijs, M. Power-law scaling in the brain surface electric potential. *PLoS Comput. Biol.* **5**, e1000609 (2009). 20019800 10.1371/journal.pcbi.1000609 PMC2787015

[25] Donoghue, T. et al. Parameterizing neural power spectra into periodic and aperiodic components. *Nat. Neurosci.* **23**, 1655–1665 (2020). 33230329 10.1038/s41593-020-00744-x PMC8106550

[26] Hohlefeld, F. U. et al. Long-range temporal correlations in the subthalamic nucleus of patients with Parkinson’s disease. *Eur. J. Neurosci.* **36**, 2812–2821 (2012). 22985199 10.1111/j.1460-9568.2012.08198.x

[27] Gao, R., Peterson, E. J. & Voytek, B. Inferring synaptic excitation/inhibition balance from field potentials. *Neuroimage* **158**, 70–78 (2017). 28676297 10.1016/j.neuroimage.2017.06.078

[28] Albin, R. L., Young, A. B. & Penney, J. B. The functional anatomy of basal ganglia disorders. *Trends Neurosci.* **12**, 366–375 (1989). 2479133 10.1016/0166-2236(89)90074-x

[29] DeLong, M. R. Primate models of movement disorders of basal ganglia origin. *Trends Neurosci.* **13**, 281–285 (1990). 1695404 10.1016/0166-2236(90)90110-v

[30] Wiest, C. et al. The aperiodic exponent of subthalamic field potentials reflects excitation/inhibition balance in Parkinsonism. *Elife* **12**, 10.7554/eLife.82467 (2023). 10.7554/eLife.82467 PMC10005762 36810199

[31] Clark, D. L. et al. Aperiodic subthalamic activity predicts motor severity and stimulation response in Parkinson disease. *Parkinsonism Relat D* **110**, 10.1016/j.parkreldis.2023.105397 (2023). 10.1016/j.parkreldis.2023.105397 37060621

[32] Busch, J. L. et al. Single threshold adaptive deep brain stimulation in Parkinson’s disease depends on parameter selection, movement state and controllability of subthalamic beta activity. *Brain Stimul.* **17**, 125–133 (2024). 38266773 10.1016/j.brs.2024.01.007

[33] He, S. et al. Beta-triggered adaptive deep brain stimulation during reaching movement in Parkinson’s disease. *Brain* **146**, 5015–5030 (2023). 37433037 10.1093/brain/awad233 PMC10690014

[34] Johnson, L. A. et al. Closed-loop deep brain stimulation effects on Parkinsonian motor symptoms in a non-human primate—is beta enough?. *Brain Stimul.* **9**, 892–896 (2016). 27401045 10.1016/j.brs.2016.06.051 PMC5143196

[35] Wingeier, B. et al. Intra-operative STN DBS attenuates the prominent beta rhythm in the STN in Parkinson’s disease. *Exp. Neurol.* **197**, 244–251 (2006). 16289053 10.1016/j.expneurol.2005.09.016

[36] Giannicola, G. et al. Subthalamic local field potentials after seven-year deep brain stimulation in Parkinson’s disease. *Exp. Neurol.* **237**, 312–317 (2012). 22735488 10.1016/j.expneurol.2012.06.012

[37] Hanrahan, S. J. et al. Long-term task- and dopamine-dependent dynamics of subthalamic local field potentials in Parkinson’s disease. *Brain Sci.* **6**, 10.3390/brainsci6040057 (2016). 10.3390/brainsci6040057 PMC5187571 27916831

[38] Neumann, W.-J. et al. Long term correlation of subthalamic beta band activity with motor impairment in patients with Parkinson’s disease. *Clin. Neurophysiol.* **128**, 2286–2291 (2017). 29031219 10.1016/j.clinph.2017.08.028 PMC5779610

[39] Cummins, D. D. et al. Chronic sensing of subthalamic local field potentials: comparison of first and second generation implantable bidirectional systems within a single subject. *Front. Neurosci.* **15**, 10.3389/FNINS.2021.725797 (2021). 10.3389/fnins.2021.725797 PMC8382799 34447294

[40] Fasano, A. et al. Subthalamic nucleus local field potential stability in patients with Parkinson’s disease. *Neurobiol. Dis.* **199**, 106589 (2024). 38969232 10.1016/j.nbd.2024.106589

[41] Belova, E. M., Semenova, U., Gamaleya, A. A., Tomskiy, A. A. & Sedov, A. Voluntary movements cause beta oscillations increase and broadband slope decrease in the subthalamic nucleus of Parkinsonian patients. *Eur. J. Neurosci.* **53**, 2205–2213 (2021). 32141151 10.1111/ejn.14715

[42] Anderson, R. W. et al. Lack of progression of beta dynamics after long-term subthalamic neurostimulation. *Ann. Clin. Transl. Neurol.* **8**, 2110–2120 (2021). 34636182 10.1002/acn3.51463 PMC8607445

[43] Anidi, C. et al. Neuromodulation targets pathological not physiological beta bursts during gait in Parkinson’s disease. *Neurobiol. Dis.* **120**, 107–117 (2018). 30196050 10.1016/j.nbd.2018.09.004 PMC6422345

[44] Connolly, A. T. et al. Modulations in oscillatory frequency and coupling in globus pallidus with increasing Parkinsonian severity. *J. Neurosci.* **35**, 6231–6240 (2015). 25878293 10.1523/JNEUROSCI.4137-14.2015 PMC4397612

[45] Strelow, J. N. et al. Local field potential-guided contact selection using chronically implanted sensing devices for deep brain stimulation in Parkinson’s disease. *Brain Sci.* **12**, 10.3390/brainsci12121726 (2022). 10.3390/brainsci12121726 PMC9776002 36552185

[46] Thenaisie, Y. et al. Principles of gait encoding in the subthalamic nucleus of people with Parkinson’s disease. *Sci. Transl. Med* **14**, eabo1800 (2022). 36070366 10.1126/scitranslmed.abo1800

[47] Zhang, G. et al. Neurophysiological features of STN LFP underlying sleep fragmentation in Parkinson’s disease. *J. Neurol. Neurosurg. Psychiatry*10.1136/jnnp-2023-331979 (2024). 38724231 10.1136/jnnp-2023-331979 PMC7616489

[48] Darmani, G. et al. Long-term recording of subthalamic aperiodic activities and beta bursts in Parkinson’s disease. *Mov. Disord.* **38**, 232–243 (2023). 36424835 10.1002/mds.29276

[49] Liu, X. et al. Subthalamic nucleus input-output dynamics are correlated with Parkinson’s burden and treatment efficacy. *NPJ Parkinsons Dis.* **10**, 117 (2024). 38879564 10.1038/s41531-024-00737-8 PMC11180194

[50] Martin, S. et al. Differential contributions of subthalamic beta rhythms and 1/f broadband activity to motor symptoms in Parkinson’s disease. *NPJ Parkinsons Dis.* **4**, 32 (2018). 30417084 10.1038/s41531-018-0068-y PMC6218479

[51] Wen, H. & Liu, Z. Separating fractal and oscillatory components in the power spectrum of neurophysiological signal. *Brain Topogr.* **29**, 13–26 (2016). 26318848 10.1007/s10548-015-0448-0 PMC4706469

[52] Gerster, M. et al. Separating neural oscillations from aperiodic 1/f activity: challenges and recommendations. *Neuroinformatics* **20**, 991–1012 (2022). 35389160 10.1007/s12021-022-09581-8 PMC9588478

[53] Priori, A. et al. Rhythm-specific pharmacological modulation of subthalamic activity in Parkinson’s disease. *Exp. Neurol.* **189**, 369–379 (2004). 15380487 10.1016/j.expneurol.2004.06.001

[54] Cheyne, D. O. MEG studies of sensorimotor rhythms: a review. *Exp. Neurol.* **245**, 27–39 (2013). 22981841 10.1016/j.expneurol.2012.08.030

[55] Mathiopoulou, V. et al. Modulation of subthalamic beta oscillations by movement, dopamine, and deep brain stimulation in Parkinson’s disease. *NPJ Parkinsons Dis.* **10**, 77 (2024). 38580641 10.1038/s41531-024-00693-3 PMC10997749

[56] van Wijk, B. C., de Bie, R. M. & Beudel, M. A systematic review of local field potential physiomarkers in Parkinson’s disease: from clinical correlations to adaptive deep brain stimulation algorithms. *J. Neurol.* **270**, 1162–1177 (2023). 36209243 10.1007/s00415-022-11388-1 PMC9886603

[57] Alagapan, S. et al. Cingulate dynamics track depression recovery with deep brain stimulation. *Nature* **622**, 130–138 (2023). 37730990 10.1038/s41586-023-06541-3 PMC10550829

[58] Merk, T. *et al*. Machine learning based brain signal decoding for intelligent adaptive deep brain stimulation. *Exp. Neurol.* **351**, 10.1016/J.EXPNEUROL.2022.113993 (2022). 10.1016/j.expneurol.2022.113993 PMC10521329 35104499

[59] Bush, A., Zou, J. F., Lipski, W. J., Kokkinos, V. & Richardson, R. M. Aperiodic components of local field potentials reflect inherent differences between cortical and subcortical activity. *Cereb. Cortex* **34**, bhae186 (2024). 38725290 10.1093/cercor/bhae186 PMC11082477

[60] Hammer, L. H., Kochanski, R. B., Starr, P. A. & Little, S. Artifact characterization and a multipurpose template-based offline removal solution for a sensing-enabled deep brain stimulation device. *Stereotact. Funct. Neurosurg.* **100**, 168–183 (2022). 35130555 10.1159/000521431 PMC9064887

[61] Sanger, Z. T. et al. Neural signal data collection and analysis of Percept™ PC BrainSense recordings for thalamic stimulation in epilepsy. *J. neural Eng.* **21**, 012001 (2024). 10.1088/1741-2552/ad1dc3 PMC11299490 38211344

[62] Stam, M. J. et al. A comparison of methods to suppress electrocardiographic artifacts in local field potential recordings. *Clin. Neurophysiol.* **146**, 147–161 (2023). 36543611 10.1016/j.clinph.2022.11.011

[63] Stanslaski, S. et al. Sensing data and methodology from the Adaptive DBS Algorithm for Personalized Therapy in Parkinson’s Disease (ADAPT-PD) clinical trial. *NPJ Parkinsons Dis.* **10**, 174 (2024). 39289373 10.1038/s41531-024-00772-5 PMC11408616

[64] Feldmann, L. K. et al. Toward therapeutic electrophysiology: beta-band suppression as a biomarker in chronic local field potential recordings. *npj Parkinson’s Dis.* **8**, 44 (2022). 35440571 10.1038/s41531-022-00301-2 PMC9018912

[65] Fornacon-Wood, I. et al. Understanding the differences between bayesian and frequentist statistics. *Int. J. Radiat. Oncol.* **112**, 1076–1082 (2022). 10.1016/j.ijrobp.2021.12.011 35286881

[66] Kruschke, J. K. Bayesian estimation supersedes the test. *J. Exp. Psychol. Gen.* **142**, 573–603 (2013). 22774788 10.1037/a0029146

[67] Gelman, A. Prior distributions for variance parameters in hierarchical models(Comment on an Article by Browne and Draper). *Bayesian Anal.* **1**, 515–533 (2006).

[68] Kenny, D. A. & Judd, C. M. Consequences of violating the independence assumption in analysis of variance. *Psychol. Bull.* **99**, 422–431 (1986).

[69] Goyal, A. et al. The development of an implantable deep brain stimulation device with simultaneous chronic electrophysiological recording and stimulation in humans. *Biosens. Bioelectron.* **176**, 112888 (2021). 33395569 10.1016/j.bios.2020.112888 PMC7953342

[70] Jimenez-Shahed, J. Device profile of the percept PC deep brain stimulation system for the treatment of Parkinson’s disease and related disorders. *Expert Rev. Med. Devices* **18**, 319–332 (2021). 33765395 10.1080/17434440.2021.1909471

[71] Thenaisie, Y. et al. Towards adaptive deep brain stimulation: clinical and technical notes on a novel commercial device for chronic brain sensing. *J. Neural Eng.* **18**, 10.1088/1741-2552/AC1D5B (2021). 10.1088/1741-2552/ac1d5b 34388744

[72] Swinnen, B. E. et al. Pitfalls and practical suggestions for using local field potential recordings in DBS clinical practice and research. *J. Neural Eng.* **22**, (2025). 10.1088/1741-2552/adaeee 39870045

[73] Cooper, S. E., Noecker, A. M., Abboud, H., Vitek, J. L. & McIntyre, C. C. Return of bradykinesia after subthalamic stimulation ceases: relationship to electrode location. *Exp. Neurol.* **231**, 207–213 (2011). 21736878 10.1016/j.expneurol.2011.06.010 PMC3375109

[74] Steiner, L. A. et al. Subthalamic beta dynamics mirror Parkinsonian bradykinesia months after neurostimulator implantation. *Mov. Disord.* **32**, 1183–1190 (2017). 28639263 10.1002/mds.27068 PMC5575541

[75] Lio, G., Thobois, S., Ballanger, B., Lau, B. & Boulinguez, P. Removing deep brain stimulation artifacts from the electroencephalogram: Issues, recommendations and an open-source toolbox. *Clin. Neurophysiol.* **129**, 2170–2185 (2018). 30144660 10.1016/j.clinph.2018.07.023

[76] Oostenveld, R., Fries, P., Maris, E. & Schoffelen, J. M. FieldTrip: open source software for advanced analysis of MEG, EEG, and invasive electrophysiological data. *Comp. Intell. Neurosci.* **2011**, 10.1155/2011/156869 (2011). 10.1155/2011/156869 PMC3021840 21253357

[77] Koo, T. K. & Li, M. Y. A Guideline of selecting and reporting intraclass correlation coefficients for reliability research. *J. Chiropr. Med.* **15**, 155–163 (2016). 27330520 10.1016/j.jcm.2016.02.012 PMC4913118

