# Supplementary material for: Long-term consistency of aperiodic and periodic physiomarkers in subthalamic local field potentials in Parkinson’s disease
Source: NPJ Parkinsons Dis. 2025 Jul 10;11:204. doi: 10.1038/s41531-025-01053-5 (PMC12246130; doi:10.1038/s41531-025-01053-5)
Supplement: Supplementary file 1 — LFP_Consistency_Supplementary Materials_Revised [file 41531_2025_1053_MOESM1_ESM.pdf]

Figure S1. Local field potential (LFP) recordings in time- and frequency domain of the left and right subthalamic nucleus (STN) of all participants during Visit 1 and ON-DBS. The blue and orange lines in the upper panels visualize 10 seconds of the filtered LFP signal in the time domain. The lower panels demonstrate the decomposition of the LFP recording into periodic and aperiodic signal components by means of the Fitting Oscillations & One Over F (FOOOF)[25] algorithm: the power spectral density (PSD) (black line), FOOOF-fitted PSD (red line), and the aperiodic component of the PSD (dashed blue line).

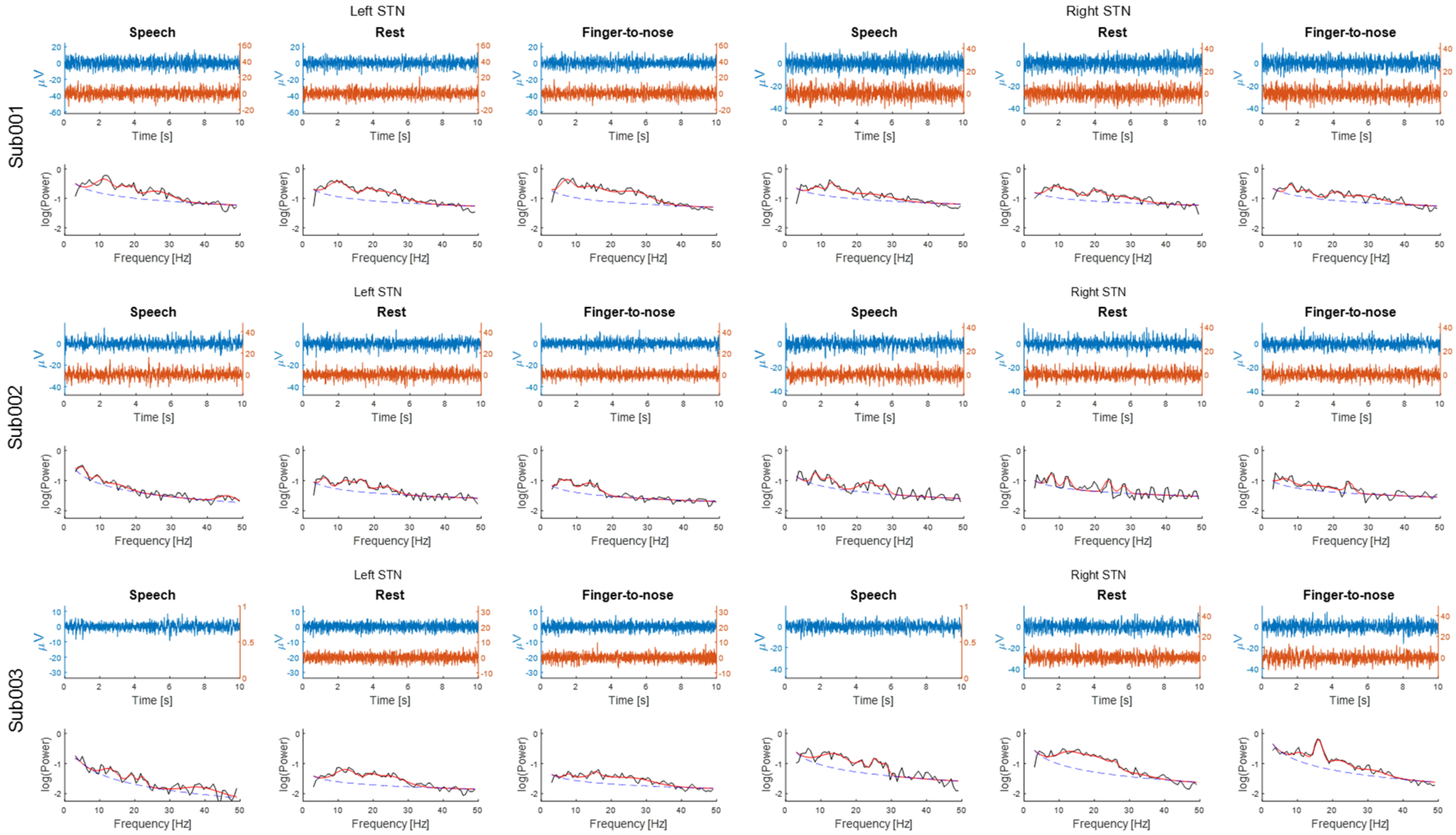

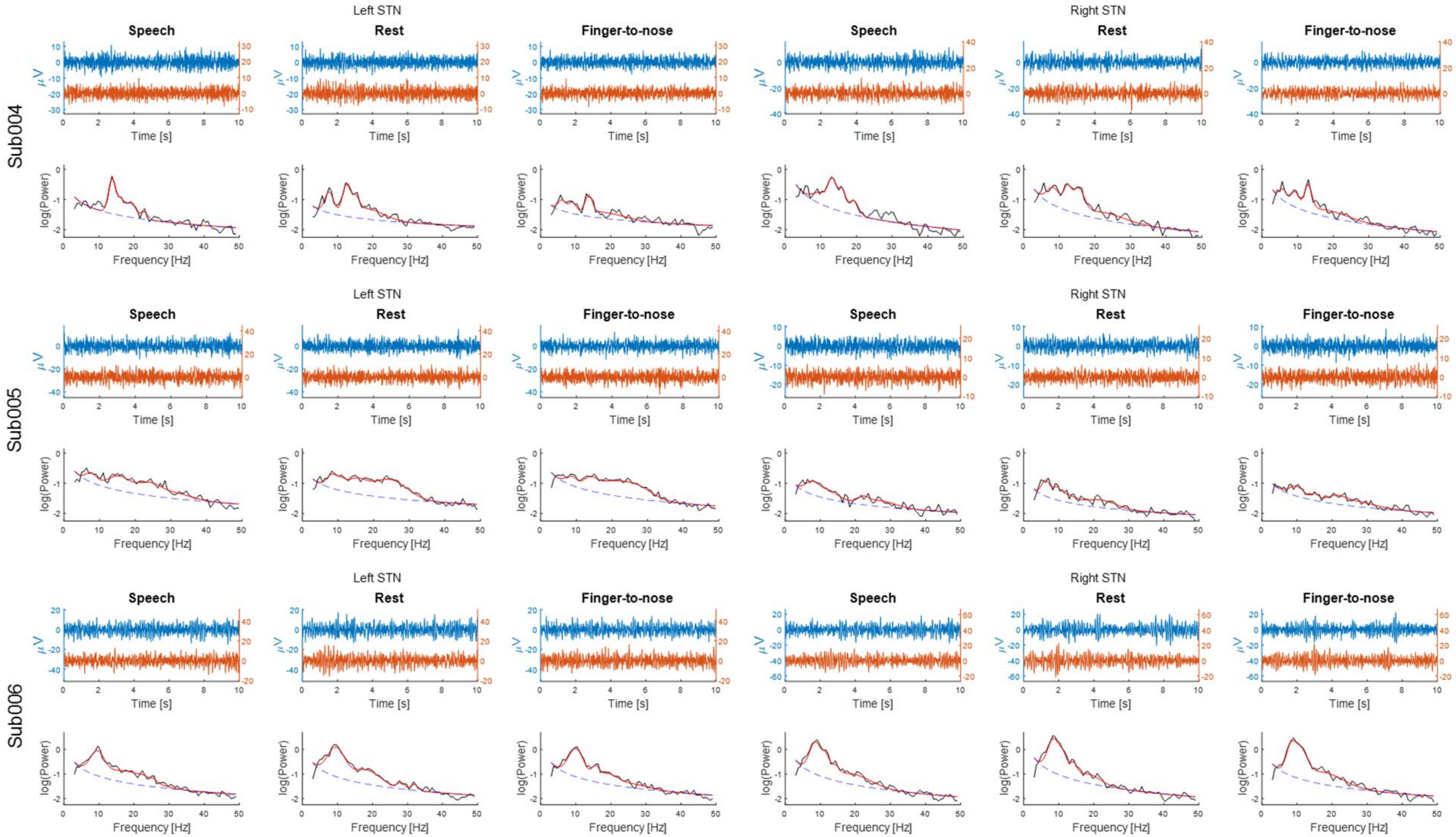

Left STN
Right STN
Speech
Rest
Finger-to-nose
Sub004
Sub005
Sub006
μV
Time [s]
log(Power)
Frequency [Hz]

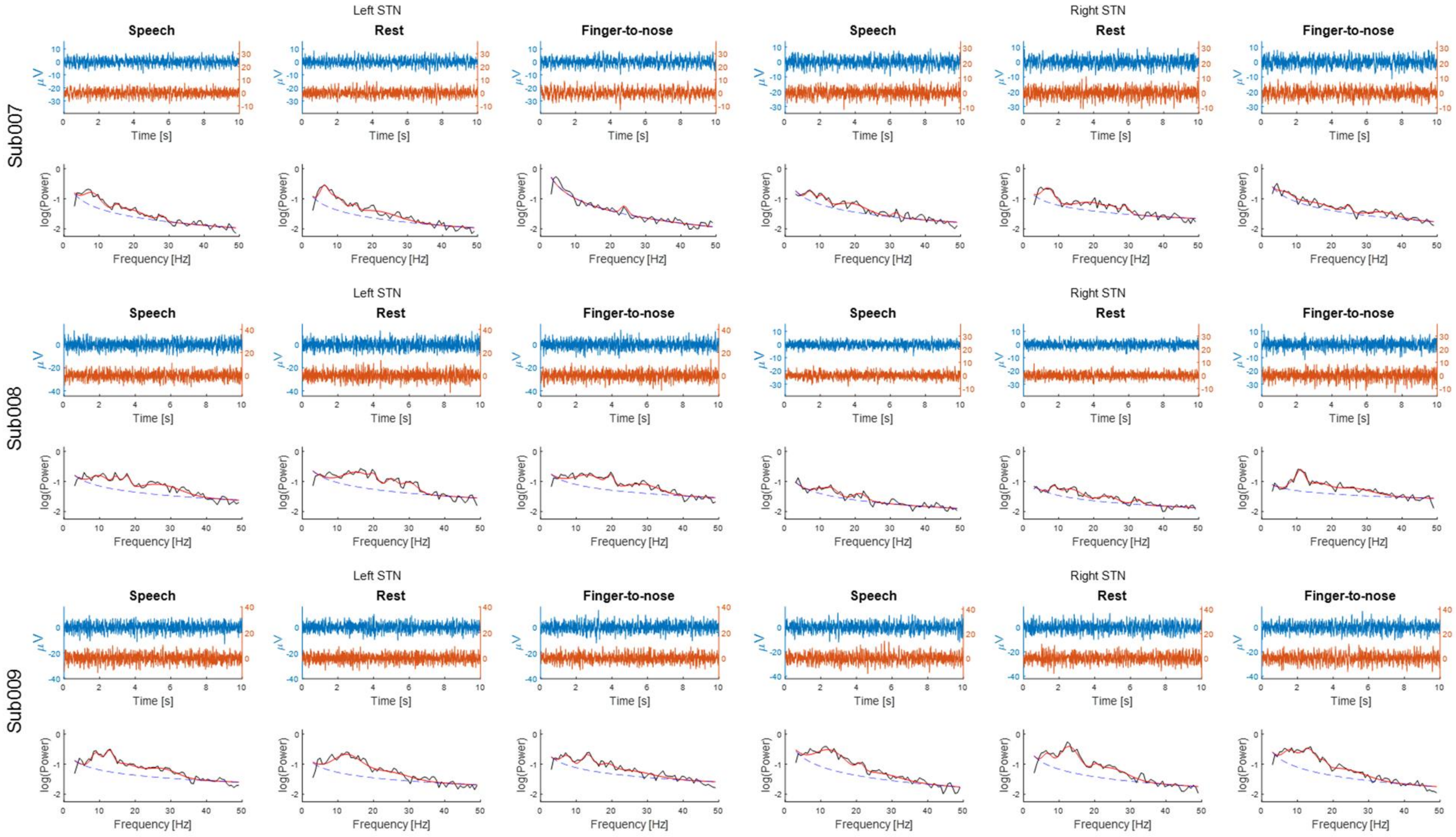

Left STN
Right STN
Speech
Rest
Finger-to-nose
Sub007
Sub008
Sub009
μV
Time [s]
log(Power)
Frequency [Hz]

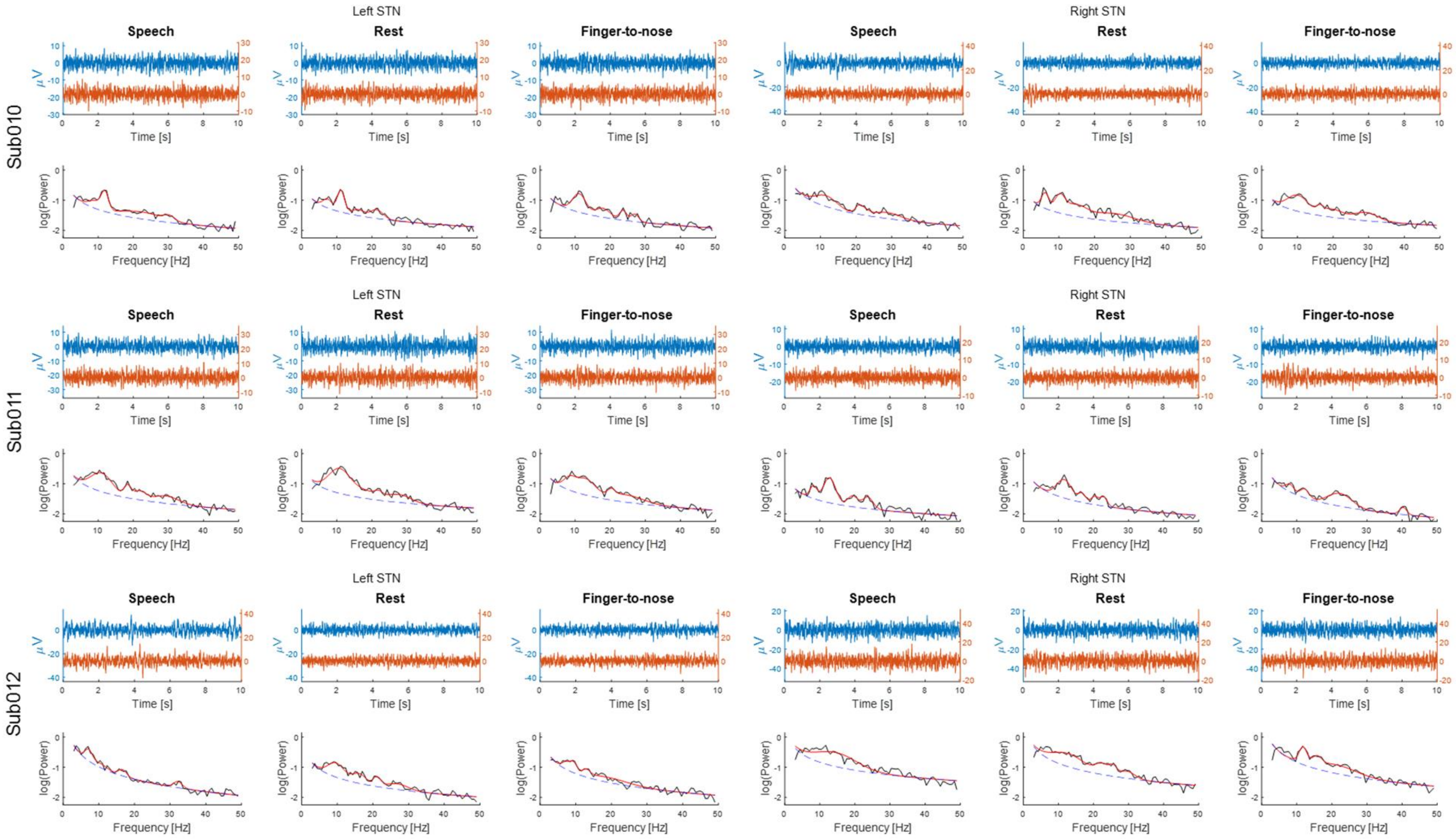

Left STN
Right STN
Speech
Rest
Finger-to-nose
Sub010
Sub011
Sub012
μV
Time [s]
log(Power)
Frequency [Hz]

Table S1. Group means and standard deviations of the log-transformed aperiodic offset and exponent determined via FOOOF. Results are presented separately per visit, task and stimulation condition.

Offset [mean ± sd]	OFF-DBS	Rest	Visit 1	-0.86 ± 0.32	Exponent [mean ± sd]	OFF-DBS	Rest	Visit 1	0.60 ± 0.17
			Visit 2	-0.87 ± 0.34				Visit 2	0.60 ± 0.19
		Finger-to-nose	Visit 1	-0.80 ± 0.30			Finger-to-nose	Visit 1	0.61 ± 0.16
			Visit 2	-0.87 ± 0.32				Visit 2	0.56 ± 0.20
		Speech	Visit 1	-0.79 ± 0.21			Speech	Visit 1	0.62 ± 0.11
			Visit 2	-0.84 ± 0.29				Visit 2	0.59 ± 0.16
	ON-DBS	Rest	Visit 1	-0.52 ± 0.36		ON-DBS	Rest	Visit 1	0.74 ± 0.25
			Visit 2	-0.50 ± 0.35				Visit 2	0.77 ± 0.21
		Finger-to-nose	Visit 1	-0.39 ± 0.41			Finger-to-nose	Visit 1	0.80 ± 0.29
			Visit 2	-0.39 ± 0.34				Visit 2	0.84 ± 0.22
		Speech	Visit 1	-0.29 ± 0.29			Speech	Visit 1	0.88 ± 0.23
			Visit 2	-0.20 ± 0.55				Visit 2	0.96 ± 0.40

Table S2. Group medians and interquartile ranges of the peak widths and (not log-transformed) power of all consistent beta peaks. Results are presented separately per visit, task and stimulation condition.

Width<br>all consistent peaks (# 45)<br>[median [Q1, Q3] Hz]	OFF-DBS	Rest	Visit 1	4.71 [2.00, 7.32]
			Visit 2	5.27 [2.07, 7.80]
Power<br>all consistent peaks (# 45)<br>[median [Q1, Q3] µV]	OFF-DBS	Rest	Visit 1	0.33 [0.18, 0.71]
			Visit 2	0.32 [0.16, 0.66]
		Finger-to-nose	Visit 1	0.25 [0.15, 0.41]
			Visit 2	0.31 [0.15, 0.51]
		Speech	Visit 1	0.23 [0.13, 0.51]
			Visit 2	0.29 [0.15, 0.44]
	ON-DBS	Rest	Visit 1	0.25 [0.17, 0.56]
			Visit 2	0.25 [0.16, 0.53]
		Finger-to-nose	Visit 1	0.27 [0.17, 0.51]
			Visit 2	0.24 [0.14, 0.45]
		Speech	Visit 1	0.28 [0.15, 0.47]
			Visit 2	0.26 [0.15, 0.60]

Table S3. The frequency and percentage change of beta power after turning the stimulation ON for all peaks found in both visits during OFF-rest (45 consistent peaks in total). Beta power suppression (BPS) is expressed as percentage decrease from 'natural fluctuations', which is defined as the absolute difference in beta power between the two visits during OFF-rest (corresponding to 100% BPS). Determination of the most stimulation-responsive beta peak was based on the peak showing the largest suppression of beta power (i.e. highest BPS value) after turning the stimulation ON, averaged over both visits (mean BPS). Per hemisphere, the selected beta peak is indicated in **Bold** font. Cases in which the frequency of the most stimulation-responsive beta peak for the left and right hemisphere differed less than 2.5 Hz are indicated in green color. BPS = beta power suppression; L = left; R = right.

	Peak #1				Peak #2				Peak #3			
Hemisphere	Peak frequency [Hz]	BPS Visit 1 [%]	BPS Visit 2 [%]	Mean BPS [%]	Peak frequency [Hz]	BPS Visit 1 [%]	BPS Visit 2 [%]	Mean BPS [%]	Peak frequency [Hz]	BPS Visit 1 [%]	BPS Visit 2 [%]	Mean BPS [%]
Sub 1-L	**15.0**	**-175.5**	**-293.1**	**-234.3**	20.6	-2851.2	-2346.5	-2598.9				
Sub 1-R	**15.5**	**481.6**	**564.8**	**523.2**	22.8	223.3	205.1	214.2				
Sub 2-L	**22.1**	**-19.4**	**106.5**	**43.5**								
Sub 2-R	22.5	-190.9	139.5	-25.7	**31.4**	**-263.6**	**226.5**	**-18.5**				
Sub 3-L	**21.2**	**-126.3**	**-3.0**	**-64.7**								
Sub 3-R	**14.3**	**28.6**	**115.3**	**72.0**	21.5	14.7	71.6	43.1				
Sub 4-L	14.6	-78.1	17.1	-30.5	**18.7**	**-23.8**	**60.8**	**18.5**				
Sub 4-R	14.9	47.1	133.9	90.5	**20.6**	**69.1**	**160.0**	**114.6**				
Sub 5-L	26.3	-620.4	-18.7	-319.6	**31.6**	**-352.9**	**159.4**	**-96.8**				
Sub 5-R	20.0	102.4	145.6	124.0	**24.1**	**1145.3**	**884.9**	**1015.1**	28.8	420.3	498.0	459.1

Sub 6-L	**21.2**	**198.6**	**231.3**	**214.9**								
Sub 6-R	**18.4**	**69.3**	**24.2**	**46.8**								
Sub 7-L	16.0	-543.2	-99.7	-321.5	20.0	-269.3	-56.8	-163.1	**23.3**	**-143.4**	**34.6**	**-54.4**
Sub 7-R	**19.0**	**-249.6**	**-140.0**	**-194.8**								
Sub 8-L	**30.4**	**-319.2**	**-260.8**	**-290.0**								
Sub 8-R	13.5	-17.7	154.4	68.4	18.5	219.7	82.8	151.2	**24.9**	**218.8**	**261.6**	**240.2**
Sub 9-L	15.1	-88.4	-471.1	-279.8	**23.3**	**79.5**	**176.6**	**128.1**				
Sub 9-R	**22.4**	**679.1**	**922.1**	**800.6**								
Sub 10-L	**15.2**	**179.9**	**253.8**	**216.9**	31.7	91.0	183.6	137.3				
Sub 10-R	14.7	-151.6	-519.7	-335.6	**18.2**	**-199.9**	**-146.7**	**-173.3**				
Sub 11-L	14.6	72.2	-4.3	33.9	**20.9**	**254.1**	**344.5**	**299.3**				
Sub 11-R	**21.8**	**248.1**	**163.7**	**205.9**								
Sub 12-L	**14.0**	261.3	346.5	303.9	18.1	306.9	261.9	284.4	**25.8**	**4122.6**	**3701.3**	**3912.0**
Sub 12-R	14.5	-3613.6	-3055.4	-3334.5	**19.1**	**-1408.1**	**-1888.1**	**-1648.1**	26.2	-4440.8	-3449.3	-3945.0

Table S4. Group medians and interquartile ranges of the peak widths and (not log-transformed) power of the most stimulation-responsive beta peaks. Results are presented separately per visit, task and stimulation condition.

Width<br>stimulation-responsive peaks (# 24)<br>[median [Q1, Q3] Hz]	OFF-DBS	Rest	Visit 1	4.62 [2.00, 6.78]
			Visit 2	6.98 [5.42, 9.20]
Power<br>stimulation-responsive peaks (# 24)<br>[median [Q1, Q3] µV]	OFF-DBS	Rest	Visit 1	0.33 [0.18, 0.72]
			Visit 2	0.40 [0.15, 1.04]
		Finger-to-nose	Visit 1	0.24 [0.15, 0.44]
			Visit 2	0.30 [0.15, 0.58]
		Speech	Visit 1	0.22 [0.12, 0.53]
			Visit 2	0.29 [0.14, 0.65]
	ON-DBS	Rest	Visit 1	0.24 [0.15, 0.38]
			Visit 2	0.22 [0.15, 0.41]
		Finger-to-nose	Visit 1	0.24 [0.15, 0.32]
			Visit 2	0.21 [0.14, 0.34]
		Speech	Visit 1	0.23 [0.14, 0.36]
			Visit 2	0.22 [0.15, 0.51]
